# Cellular function reinstitution of offspring red blood cells cloned from the sickle cell disease patient blood post CRISPR genome editing

**DOI:** 10.1186/s13045-017-0489-9

**Published:** 2017-06-13

**Authors:** Jianguo Wen, Wenjing Tao, Suyang Hao, Youli Zu

**Affiliations:** 1 Department of Pathology and Genomic Medicine, Houston Methodist Hospital, Houston Methodist Research Institute, Houston, TX 77030 USA; 2 Department of Leukemia, The University of Texas M.D. Anderson Cancer Center, Houston, TX 77030 USA

**Keywords:** CRISPR/Cas9 genome editing, Hematopoietic stem/progenitor cell colonies, Sickle cell disease

## Abstract

**Background:**

Sickle cell disease (SCD) is a disorder of red blood cells (RBCs) expressing abnormal hemoglobin-S (HbS) due to genetic inheritance of homologous HbS gene. However, people with the sickle cell trait (SCT) carry a single allele of HbS and do not usually suffer from SCD symptoms, thus providing a rationale to treat SCD.

**Methods:**

To validate gene therapy potential, hematopoietic stem cells were isolated from the SCD patient blood and treated with CRISPR/Cas9 approach. To precisely dissect genome-editing effects, erythroid progenitor cells were cloned from single colonies of CRISPR-treated cells and then expanded for simultaneous gene, protein, and cellular function studies.

**Results:**

Genotyping and sequencing analysis revealed that the genome-edited erythroid progenitor colonies were converted to SCT genotype from SCD genotype. HPLC protein assays confirmed reinstallation of normal hemoglobin at a similar level with HbS in the cloned genome-edited erythroid progenitor cells. For cell function evaluation, in vitro RBC differentiation of the cloned erythroid progenitor cells was induced. As expected, cell sickling assays indicated function reinstitution of the genome-edited offspring SCD RBCs, which became more resistant to sickling under hypoxia condition.

**Conclusions:**

This study is an exploration of genome editing of SCD HSPCs.

**Electronic supplementary material:**

The online version of this article (doi:10.1186/s13045-017-0489-9) contains supplementary material, which is available to authorized users.

## Background

Sickle cell disease (SCD) is an inherited blood disorder caused by the point mutation 69A>T in the hemoglobin beta chain gene (*HBB*), encoding the beta globin subunit of hemoglobin-A (HbA) in normal red blood cells (RBCs). This genomic mutation of *HBB* results in expression of abnormal hemoglobin-S (HbS). RBCs of SCD patients produce HbS and lack HbA because they inherit two alleles of *HbS* gene. Cellular HbS molecules at high concentration tend to stick together and form polymers under stress conditions including hypoxia, high altitude, dehydration, and temperature changes. Polymerization of abnormal cellular HbS causes deformation of RBCs rendering them rigid and sickle- or crescent-shaped. The resulting sickle-shaped RBCs can stick in small vessel walls and break down prematurely, which induces anemia, bacterial infections, and stroke [1, 2]. Currently, allogeneic bone marrow transplant is the only potential approach to cure SCD [3, 4]. However, in clinical practice, locating a suitable donor is difficult and the allogeneic marrow transplant procedure has serious risks, including patient death [4, 5]. On the other hand, people with sickle cell trait (SCT) carry the heterozygous genotype with a single allele of both *HbS* and *HBB* genes and usually do not experience symptoms of SCD due to co-presence of normal HbA and HbS in RBCs [6]. Taking this into consideration, the therapeutic rationale to treat SCD patients can be founded on conversion of SCD to SCT genotype via genome editing of *HbS/HbS* to *HBB/HbS*, as illustrated in Additional file 1: Figure S1A.

Clustered regularly interspaced short palindromic repeats (CRISPR) and CRISPR-associated (Cas) genes were initially discovered in *Escherichia coli* [7]. In 2007, Barrangou et al. demonstrated that integrating a genome fragment of an infectious virus into its CRISPR locus conferred *Streptococcus thermophilus* resistance against a bacteriophage [8]. In 2012, Jinek et al. demonstrated the capacity of CRISPR/Cas9 system to perform RNA-programmable genome editing [9]. This approach for genome editing has been studied in a variety of organisms spanning bacteria [10], yeasts [11], *Caenorhabditis elegans* [12], *Xenopus tropicalis* [13], plants [14], Drosophila [15], zebrafish [16], and mammalian cells from mice [17], rats [18], rabbits [19], monkeys [20], and pigs [12] to humans [14].

To explore feasibility to treat SCD, Huang et al. demonstrated the utility of CRISPR/Cas9 method in genome editing *HbS* in induced pluripotent stem cells derived from SCD patients [21]. Similarly, Hoban et al. reported that genome editing of CD34+ hematopoietic stem/progenitor cells (HSPCs) from the bone marrow of a SCD patient and heterozygous *HbS* correction led to an increase in production of normal hemoglobin [22]. DeWitt et al. also demonstrated that CRISPR/Cas9 can mediate efficient gene editing for SCD [23]. In addition, the engineered zinc-finger nuclease (ZFN) approach was tested as a means to correct the *HbS* mutation in HSPCs from the SCD patient bone marrow [24].

In this study, we validated the genome editing of *HbS* using HSPCs derived from a small amount of the SCD patient peripheral blood with CRISPR/Cas9 method. Resultant erythroid progenitor cells were cloned from individual colonies of patient HSPCs post CRISPR treatment. Genome-editing status of the cloned cells was confirmed by both gene sequencing and hemoglobin protein expression. Finally, in vitro differentiation of the cloned erythroid progenitor cells was carried out, and cellular function reinstitution of the offspring RBCs was confirmed. These findings provide a solid foundation to treat SCD by genome editing of patient HSPCs using CRISPR/Cas9 approach. (Additional file 1: Figure S1).

## Methods

### Materials

All reagents were purchased from Thermo Fisher Scientific (Waltham, MA) unless otherwise stated. All oligonucleotides were synthesized by IDT (Integrated DNA Technologies, Coralville, IA).

### HEL cell cultures

Human erythroblast cell line, HEL, was purchased from the American Type Culture Collection (ATCC). Cells were grown in RPMI 1640 complete culture medium supplemented with 10% fetal bovine serum (FBS, Atlanta Biologicals, Atlanta, GA), 100 U/mL penicillin, and 100 μg/mL streptomycin [25]. HEL cells stably expressing enhanced green fluorescent protein (EGFP) were established as previously reported [26].

### Isolation of CD34+ hematopoietic stem/progenitor cells from the patient peripheral blood

A small amount of the peripheral blood (2–3 mL) was collected from SCD patients post disease diagnosis under an approved institutional review board (IRB) protocol. First, the presence of HSPCs in the peripheral blood of SCD patients was assessed by staining cells with anti-CD34, CD45, CD19, and CD2 antibodies and flow cytometry analysis. To isolate HSPCs, the MACS CD34 Progenitor Cell Isolation Kit (Miltenyi Biotec, Auburn, CA) was used. Briefly, 2–3 mL of the blood was collected, transferred to a 50-mL conical tube, and RBCs were lysed by incubation in 20 mL of RBC lysis buffer for 10 min (Biolegend, San Diego, CA). Unlysed cells were then suspended in MACS buffer and digested in collagenase IV (10 mg/ml) and 10 U/ml DNase at 37 °C for 40 min. Subsequently, cells were incubated with anti-CD34 antibody-conjugated microbeads (Miltenyi Biotech, Auburn, CA) for 30 min at 4 °C, and HSPCs were then isolated on an AutoMacs device [27]. For characterization, 5 × 10^3^ isolated HSPCs were stained with APC-conjugated anti-CD34 antibody and analyzed by flow cytometry with BD LSRII. In addition, 1 × 10^3^ HSPCs were suspended in 100 μL PBS, and cytospin slides were prepared. The cytospin slides were stained with Wright-Giemsa stain using Hema-TEK 2000 Slide Stainer (Thermo Fisher Scientific, Houston, TX) and examined under a microscope. To obtain sufficient cells for genome-editing studies, in vitro expansion was conducted by culturing HSPCs in Stemspan SFEM II supplemented with CC100 cytokine cocktail for 7 days (StemCell Technologies, Vancouver, Canada). Resultant HSPCs were counted, and the numbers were compared before and post cell expansion [28].

### Amplification and characterization of cloned erythroid progenitor cells

Half of erythroid progenitor cells cloned from individual single E-colonies on Methocult was directly used for RT-PCR genotyping and sequencing analysis. The other half was transferred to a 24-well plate containing 2 mL erythroid progenitor expansion (EPE) medium [29], composed of StemSpan SFEM II medium supplemented with 20 ng/mL erythropoietin (EPO) (PeproTech, Rocky Hill, NJ), 100 ng/mL stem cell factor (SCF) (PeproTech), 50 ng/mL insulin-like growth factor-1 (IGF-1) (PeproTech), and 2 μM dexamethasone (Sigma Aldrich, St. Louis, MO). Cloned cells were cultured for 10 days and had medium changes on days 4 and 7.

In contrast, cells cloned from single G/M-colonies were just used for RT-PCR genotyping and sequencing analysis without amplification. No further cellular protein or functional assays were performed with them because they do not produce hemoglobin or form sickle cells.

To evaluate genome-editing status of cloned cells derived from individual E- and G/M-colonies, cellular genomic DNA was prepared, and genotyping and sequencing analysis were carried out as previously described. Genome-editing efficacy in E and G/M progenitor cells was calculated.

For immunophenotyping confirmation, amplified erythroid progenitor cells (1 × 10^4^) were stained with the antibodies APC (allophycocyanin)-labeled anti-CD34, anti-CD71, anti-CD235a, anti-CD45, or FITC (fluorescein isothiocyanate)-labeled CD36 (BioLegend). Cells were also treated with DRAQ5 DNA dye (BioLegend) to stain the nuclei. Individual biomarker expression and the presence of nuclei were detected by flow cytometry (LSR II, BD Biosciences, San Jose, CA).

### Detection of cellular hemoglobin proteins by clinically viable HPLC method

Half of the amplified erythroid progenitor cells (5 × 10^4^) from individual single colonies were incubated in 1.5 mL of D-10 Wash/Diluent Solution (BioRad, Hercules, CA) for 5 min at room temperature. For high-performance liquid chromatography (HPLC) analysis, cell lysates (in 2.0-mL tubes) were loaded on a BioRad D10 HPLC instrument and analyzed [30], following standard clinical protocol in our department. For each test, 0.25 mL of erythroid progenitor cell lysates (~8300 cells/assay) was used. Peaks for HbA* that resulted from genome-engineered HBB* and HbS protein in cloned erythroid progenitor cells were detected based on HPLC retention times.

To establish standard controls for identification of HbA and HbS, blood samples from SCD patients, SCT carriers, and normal persons were collected under approved IRB protocol. Blood cells were then diluted in 1.5 mL D-10 Solution to reach a final count of 2500 to 22500 RBCs/assay. Cell lysates were prepared, and HPLC assay was performed under the previously described conditions (Additional file 1: Figure S16).

### In vitro RBC differentiation of cloned erythroid progenitor cells

To induce in vitro differentiation and maturation, the other half of amplified erythroid progenitor cells were seeded in 1 mL Erythroid Terminal Maturation (ETM) medium[21], composed of Iscove’s modified Dulbecco’s medium supplemented with 20 ng/mL EPO, 50 ng/mL insulin (Sigma), 4 U/mL heparin, 200 μg/mL holo-transferrin (Sigma), and 2% human serum albumin (Sigma). Cells were cultured for 8 days with medium change on day 4.

To confirm in vitro RBC differentiation post induction, cells (1 × 10^3^) were suspended in 100 μL PBS and nuclear-stained with Hoechst 33342 dye (3 μg/ml final concentration, Thermo Fisher Scientific) for 30 min at room temperature. Matured RBCs lacking nuclei and nucleated RBCs were detected under optical and fluorescence microscopes. In addition, cytospins of the differentiated cells were prepared, and slides were stained with Wright-Giemsa stain as previously described. RBCs at different maturation status were detected under a microscope.

### Cell sickling tests of in vitro differentiated genome-edited RBCs

For cell sickling assays, in vitro differentiated cells resuspended in 2 μL PBS were mixed with 2 μL of 2% sodium metabisulfite (Sigma) to create hypoxia condition [31]. Cells were then loaded on a glass slide and immediately covered with a cover slip. After carefully removing excess solution below the cover slip, all cover slide edges were rapidly sealed with nail varnish. Cell sickling formation was kinetically recorded at 1-min intervals for 30 min under a microscope (Olympus). For quantitative analysis, a total 500 cells were counted and the percentage of sickling cells vs. time lapse was plotted.

Supplemetary matierals are availabe as Additional file 1, available on the *Journal of Hematology & Oncology* Web Site.

## Results

### Establishment of an efficient CRISPR/Cas9 approach

To optimize genome-editing conditions, we used human erythroleukemia (HEL) cells, a cell line carrying normal *HBB/HBB* genotype [32]. First, to test the electroporation efficacy, the DNA sequence encoding sgRNA in vivo for *HBB* gene was amplified by PCR and labeled with Cy3 fluorescent reporter. The optimal electroporation conditions for HEL cells were determined (Additional file 1: Figure S3). Subsequently, to assess CRISPR efficacy, the Cas9 mRNA and DNA sequence encoding sgRNA in vivo, specific for enhanced green fluorescent protein gene (*EGFP*), were introduced by electroporation into stably EGFP-expressing HEL cells. The resultant *EGFP* gene silencing was quantified by flow cytometry (Additional file 1: Figure S4). To verify RT-PCR genotyping of cell *HbS* and *HBB* genes, standard curves were established using genomic DNA from the whole blood of SCD patients and healthy donors at different ratios (Additional file 1: Figure S5). In addition, sgRNA specific for *HBB* was also produced by in vitro transcription (Additional file 1: Figure S6) and introduced into HEL cells by electroporation with an homology-directed repair (HDR) template to convert *HBB* to *HbS*. Target genome-editing efficacy was evaluated by genotyping and compared to that induced by the DNA sequence encoding sgRNA in vivo (Additional file 1: Figure S7). Furthermore, the ratio of sgRNA to HDR template used for electroporation was optimized (Additional file 1: Figure S8). To achieve the highest genome-editing efficiency, HDR templates targeting different cutting loci in *HBB* (Additional file 1: Figure S9) and HDR templates with different lengths (Additional file 1: Figure S10) were investigated.

For further validation, we created a SCD cell model by applying the optimized CRISPR/Cas9 approach as previously described. Step-by-step genome-engineering was conducted, and the resultant SCD HEL cells carrying homozygous *HbS/HbS* genotype were cloned (Additional file 1: Figure S11A). Genotyping and sequencing analysis showed 9.6 and 9.4% genome-editing efficacy in each CRISPR step (Additional file 1: Figure S11B). Finally, to mimic disease treatment, SCD HEL cells were genome-engineered to convert *HbS/HbS* to *HBB/HbS*, corresponding to the conversion of SCD to SCT genotype (Additional file 1: Figures S12 and S13). The aforementioned validation studies demonstrated reproducibility, efficiency, and feasibility of this CRISPR/Cas9 approach for clinical studies (Additional file 1: Figure S1).

### Development of a clinically practicable approach for genome-editing therapy of SCD

**Fig. 1:**
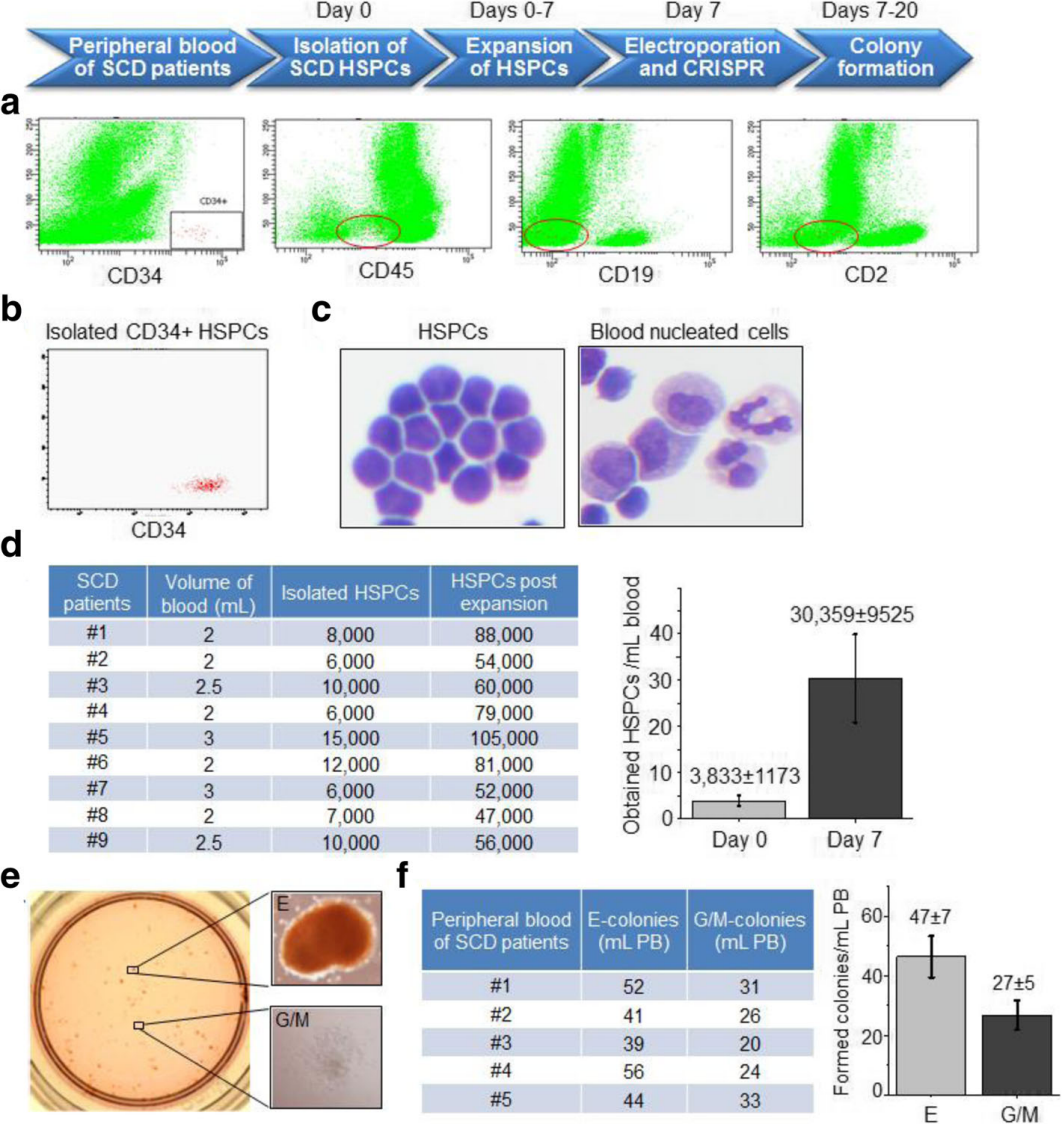
Preparation of HSPCs from the peripheral blood of SCD patients and CRISPR/Cas9 genome editing. **a** Immunophenotyping analysis of HSPCs in patient whole blood by flow cytometry. **b** Immunophenotyping of isolated CD34+ HSPCs. **c** Morphological examination of CD34+ HSPCs and CD34-blood-nucleated cells with Wright-Giemsa stain. **d** Cell counts of isolated HSPCs pre- and post-expansion. **e** Colony formation of HSPCs post CRISPR genome editing. **f** Counts of formed E- and G/M-colonies per milliliter of the patient peripheral blood

For target genome editing of *HbS* by the optimized CRISPR/Cas9 method, we used small amounts of the peripheral blood from SCD patients (2–3 mL). Immunophenotyping analysis of the patient blood revealed a scant number of HSPCs that were positive for CD34 expression, but negative for CD45, CD19, or CD2 (Fig. 1a). The HSPCs were then isolated using the MACS CD34+ Progenitor Cell Isolation Kit and confirmed by immunophenotyping analysis (Fig. 1b). For morphology evaluation, cytospin was performed with Wright-Giemsa staining, and the cells were examined under the microscope. In comparison to blood-nucleated cells, the isolated HSPCs were uniform in size and shape with very scant cytoplasm (Fig. 1c). The HSPCs were cultured for 7 days in Stemspan SFEM II medium, resulting in an average eightfold increase in cell number (Fig. 1d).

**Fig. 2:**
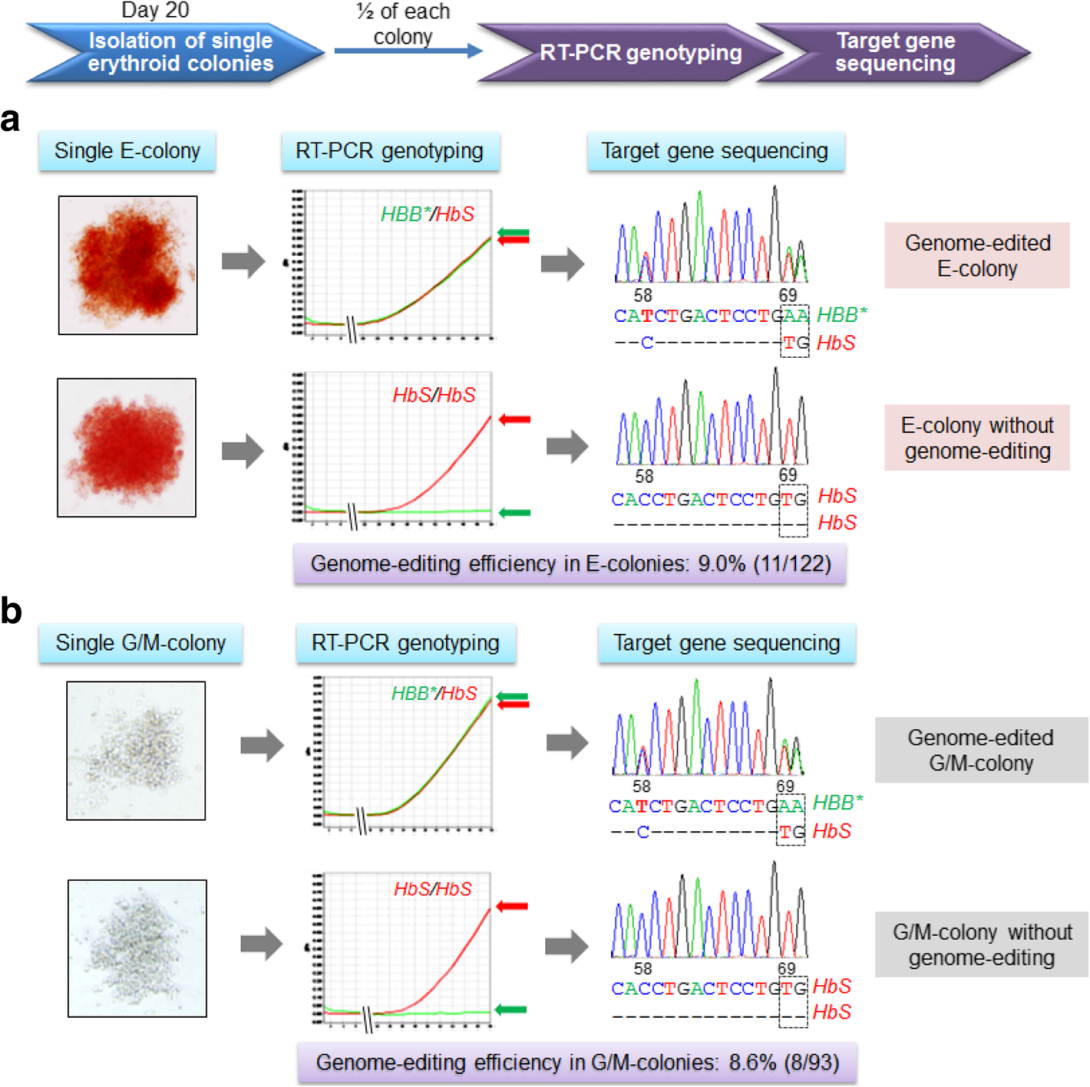
Validation of genome-editing status of cloned erythroid progenitor cells. **a** RT-PCR genotyping and sequencing analysis of cloned erythroid progenitor cells. **b** Genotyping and sequencing analysis of cloned cells from G/M-colonies derived from HSPCs of the same patient post CRISPR genome editing

For CRISPR genome editing, the HSPCs were subjected to electroporation for intracellular delivery of Cas9 mRNA, and then cultured for 6 h to allow expression of cellular Cas9 protein. Subsequently, cells were repeatedly subjected to electroporation to introduce sgRNA specifically targeting *HbS* and the 127nt HDR. To correct the V6G mutation in HbS protein, nucleotides 69T and 70G in *HbS* were substituted with 69A and 70A, respectively, to encode glutamic acid at position 6 in HBB* of the expressed HbA* protein identical to natural HbA (Additional file 1: Figure S1A). Notably, to distinguish genome-edited HBB* from natural *HBB*, the point mutations 70G>A and 58C>T, which do not alter the encoded amino acid residues, were introduced as hallmarks of HBB*. For cell colony formation, the electroporated HSPCs were seeded in Methocult H4030 medium and cultured for 13 days. The formed erythroid progenitor colonies (E-colonies), which had a light reddish color, and the granular/monocytic progenitor colonies (G/M-colonies), which appeared semi-transparent, were detected under the microscope (Fig. 1e), revealing an average 47 ± 7 E- and 27 ± 5 G/M-colonies per milliliter of the SCD patient blood (Fig. 1f). Giemsa-Wright staining showed the morphology of the erythroid progenitor cells (Additional file 1: Figure S14). To evaluate genome-editing status, genomic DNA was prepared from the half of cloned cells, and RT-PCR genotyping screening was performed using TaqMan MGB probe-reporter system specific for HBB* and *HbS* as described in “Materials” and “Methods.” Simultaneously, target gene sequencing analysis was performed; equal amounts of *HbS* and engineered HBB* containing nucleotide 69A and the tracking nucleotides 58T and 70A were detected (Fig. 2a). As an experiment internal control, homozygous *HbS* was detected in cloned cells from the same patient HSPCs that had not undergone genome editing. The overall genome-editing efficacy of HSPCs in E-colonies was 9.0%, which is similar to that detected in the validation studies of SCD HEL cell model. Moreover, according to the chromatogram of Sanger sequencing, the other allele of corrected single E-colony and G/M-colony cells does not carry indels near the cleavage site (Additional file 1: Figure S15), as the results in the corrected SCD HEL cells (Additional file 1: Figure S13). The genome-editing status of G/M-colonies was also evaluated by both RT-PCR genotyping and sequencing analysis, revealing 8.6% efficacy, which is nearly identical to that found in E-colonies derived from HSPCs from the same patient (Fig. 2b).

**Fig. 3:**
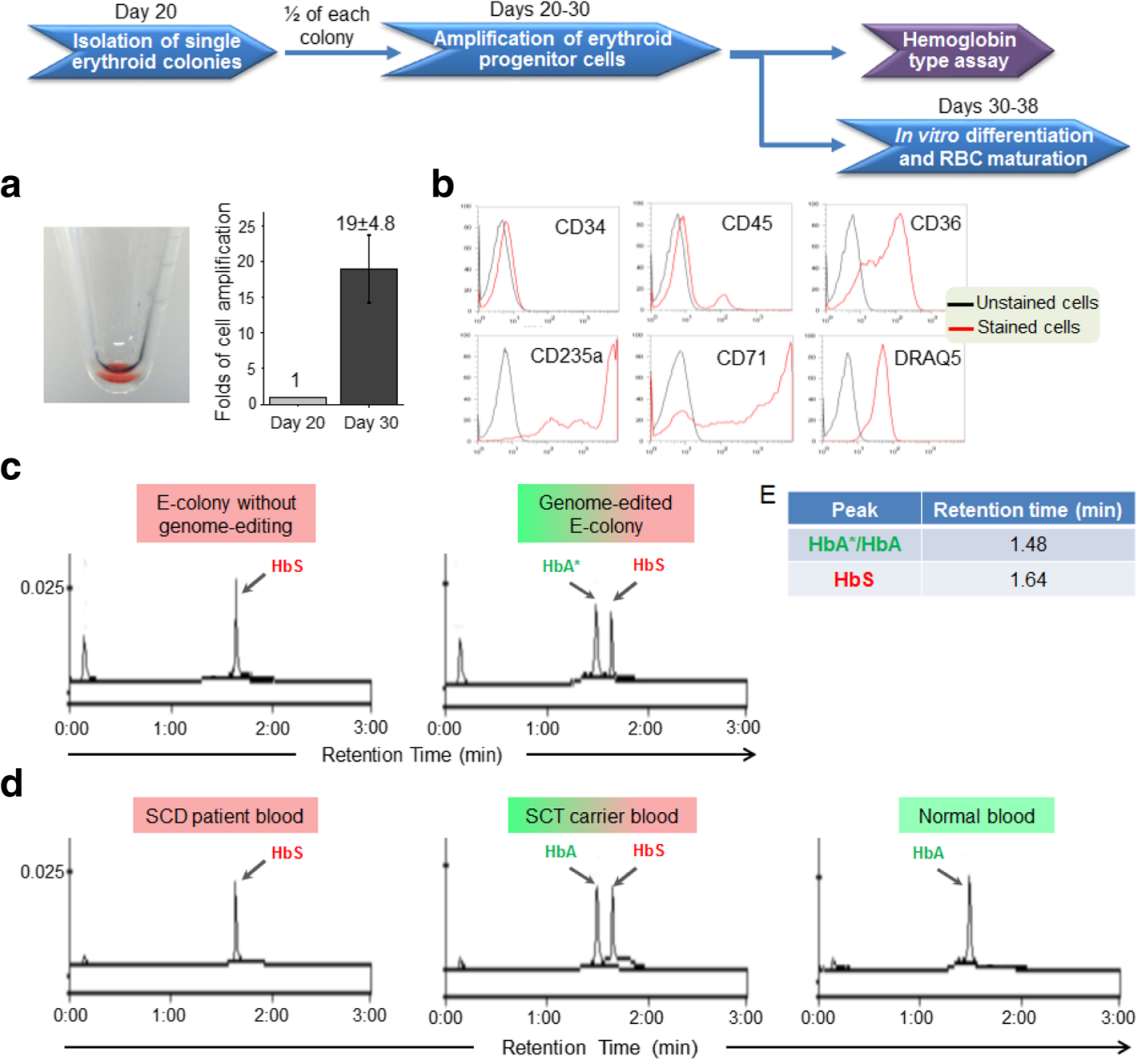
Validation of cellular hemoglobin expression. **a** Cells were cloned from single E-colonies, formed from HSPCs post CRISPR genome editing, and amplified after culturing for 10 days; *left*: pellet of amplified cells had reddish color; *right*: cell amplification. **b** Immunophenotyping analysis of cloned erythroid precursor cells by flow cytometry. **c** HPLC assay revealed a new peak for HbA* protein in the genome-edited erythroid progenitor cells (*right panel*); in contrast, a peak for HbS only was present in cloned E-colony cells that did not undergo genome editing as the experiment internal control (*left panel*). **d** Hemoglobin in the peripheral blood from SCD patient, SCT carrier, and normal person were used as HPLC assay standard controls. **e** HPLC retention times corresponding to peaks for HbA*/HbA and HbS. Notably, peaks for HbA* and natural HbA were detected at the same retention time because they have identical amino acid sequences

Subsequently, the other half of cells from single E-colonies was transferred to an erythroid progenitor medium for clonal cell amplification [29], and the cells were cultured for 10 days. The erythroid progenitor cells, cloned from single E-colonies, were amplified 19-fold on average and showed an enhanced red color, indicating synthesis of cellular hemoglobin (Fig. 3a). The amplified cells were stained with anti-CD34, CD36, CD45, CD71, and CD235a antibodies, and the nuclei were stained with DRAQ-5 dye. Flow cytometry demonstrated that the cloned cells had an immunophenotyping profile consistent with erythroid progenitor cells; in particular, they expressed CD36, CD235a, and CD71; lacked CD34 and CD45; and possessed nuclei (Fig. 3b) [21].

To investigate changes in cellular protein expression, the cloned cells were lysed, and hemoglobin types were assessed by a clinically viable HPLC method (8.3 × 10^3^ cells/test) as described in “Materials” and “Methods” (Additional file 1: Figure S15) [30]. Due to genome editing resulting in heterozygous HBB*/HbS, a new peak for HbA* protein was detected in the cloned erythroid progenitor cells at a similar level to residual HbS protein (Fig. 3c). In the experiment internal control, the cloned cells from the same patient HSPCs that did not undergo genome editing expressed the HbS protein only. For further comparison, the HPLC elution profiles of blood samples from normal people, SCT carriers, and SCD patients are shown in Fig. 3d. Notably, although HBB* contains two different nucleotides as compared to *HBB*, it encodes the same amino acid residues, and thus, HbA* appears at the same retention time with natural HbA in HPLC assay.

**Fig. 4:**
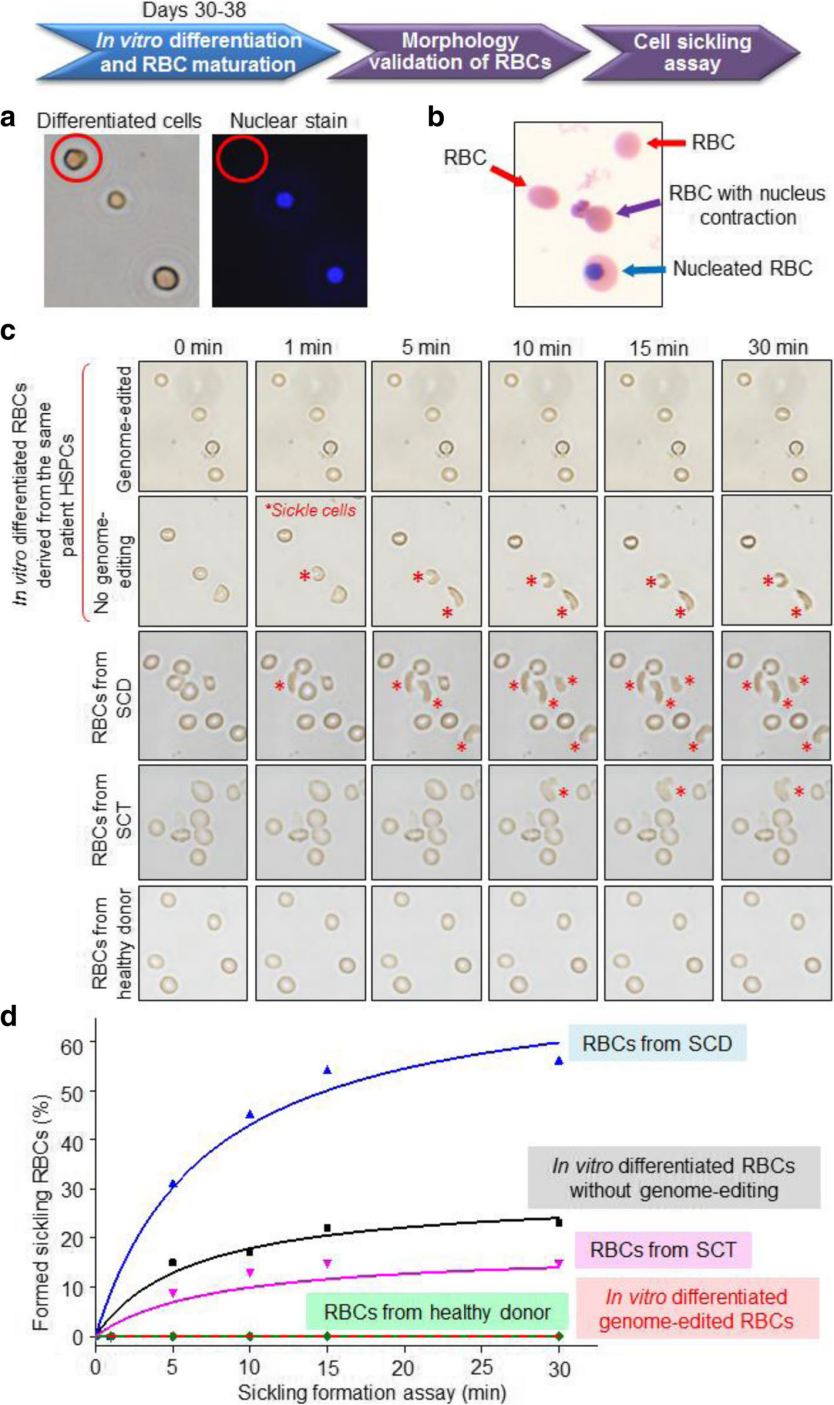
Cellular function reinstitution of genome-edited RBCs. **a** For cellular function study, in vitro RBC differentiation of cloned erythroid progenitor cells was induced. Nuclei of the resultant cells were initially stained with Hoechst dye and examined under microscope, demonstrating induced in vitro RBC differentiation and maturation. **b** Cytospin of resultant cells with Wright-Giemsa staining confirmed presence of mature RBCs (no nuclei), RBC with nucleus contraction, and nucleated RBC. **c** Cell sickling assays of in vitro differentiated RBCs with or without genome editing, and RBCs from the SCD, SCT, and healthy donor blood demonstrated that CRISPR genome editing of patient HSPCs resulted in function reinstitution of the cloned offspring RBCs, which became more resistant to hypoxia. **d** Time course of sickling cell formation (%). The data are from the average of three clones



For cellular function study, the amplified erythroid progenitor cells were further cultured in erythroid terminal maturation medium [21] to induce in vitro differentiation and RBC maturation as described in “Materials” and “Methods.” Nuclear staining with Hoechst fluorescent dye was initially used to assess the maturation states of cells. Microscopic examination revealed presence of nucleated RBCs and mature RBCs that did not have nuclei (Fig. 4a). In addition, cytospin with Wright-Giemsa staining confirmed in vitro RBC differentiation at different maturation stages, including nucleated RBCs, RBCs with nuclear contraction, and mature RBCs with no nuclei (Fig. 4b) [33]. To evaluate cellular function changes resulting from genome editing, cell sickling tests were performed. To induce hypoxia, in vitro differentiated RBCs were suspended in sodium metabisulfite buffer, loaded on a glass slide, and immediately covered with a cover slip. The time course of the resultant sickle cell formation was recorded under a microscope at 1-min intervals for 30 min. As shown in Fig. 4c and Additional files 2, 3, and 4: Movies S1–S3, the genome-edited RBCs were more resistant to hypoxia and did not display sickling formation. In contrast, under the same hypoxia condition, sickle cell formation was observed in the in vitro differentiated RBCs from the same patient HSPCs that did not undergo genome editing, i.e., experiment internal control. The peripheral blood from SCD, SCT, and healthy donors were also tested as control. Some RBCs from SCT sickled in the test but much more sickled RBCs presented in the specimen from SCD (Fig. 4c). For quantitative analysis, 500 RBCs from each specimen were examined and the percentage of sickle cells vs. time lapse was calculated (Fig. 4d). These findings demonstrated that genome editing of HSPCs from a SCD patient reinstituted cellular function of the offspring RBCs, eliminated sickling formation under hypoxia, and confirmed feasibility of gene therapy to treat SCD. Notably, because a number of the in vitro differentiated RBCs contained nuclei (Figs. 4a, b) and their cellular shape could not change, the RBCs in the experiment internal control group showed a lower percentage of sickle cell formation than that observed for peripheral blood RBCs from the SCD patient under the same hypoxia conditions (Fig. 4d). Thus, the sickling of in vitro differentiated RBCs from the genome-edited SCD patient HSPCs may also be underestimated.

**Additional file 2: Movie S1.** Sickling assay of genome-edited RBCs. (WMV 4447 kb)




**Additional file 3: Movie S2.** Sickling assay of RBCs without genome editing. (WMV 4213 kb)




**Additional file 4: Movie S3.** Sickling assay of RBCs from the SCD patient blood. (WMV 4629 kb)


### Validation of a Cas9/sgRNA ribonucleoprotein system for SCD genome editing

**Fig. 5:**
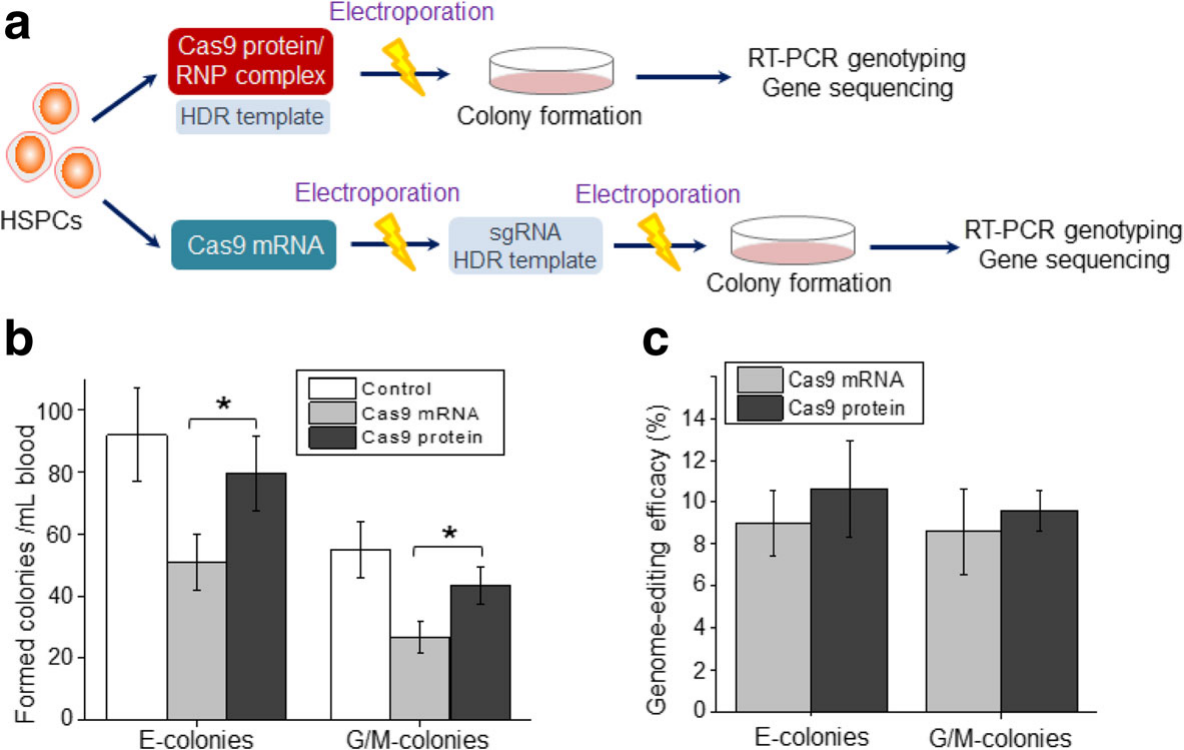
Comparison of CRISPR genome editing using Cas9 protein or Cas9 mRNA. **a** Scheme depicting CRISPR using Cas9 protein with a single electroporation vs. Cas9 mRNA with repeated electroporation steps. **b** E- and G/M-colonies formed on Methocult from unmanipulated HSPCs, CRISPR genome-edited HSPCs mediated with Cas9 protein, and CISPR genome-edited HSPCs mediated with Cas9 mRNA. *p ≤ 0.05. **c** The efficacy of CRISPR genome editing mediated with Cas9 protein or Cas9 mRNA

To reduce the number of electroporation steps, the newly reported Cas9/gRNA ribonucleoprotein (RNP) delivery system was tested and compared to the method used in this study (Fig. 5a). A genome-editing RNP complex was formulated by mixing the Cas9 protein with two synthetic RNA oligonucleotides, a CRISPR targeting RNA (crRNA) duplexed to a trans-activating crRNA (tracrRNA) as described in “Materials” and “Methods.” The formed Cas9/RNP complex and HDR template, used to convert *HbS* to HBB*, were introduced by electroporation into HSPCs isolated from the SCD patient blood. CRISPR-treated HSPCs were then cultured to form cell colonies, and genome-editing status of the resultant cells cloned from individual colonies was evaluated by RT-PCR genotyping and gene sequencing. Single-step electroporation with Cas9/RNP complex resulted in a significant improvement in cell colony formation of HSPCs, namely 56 and 73% increase in formation of E- and G/M-colonies, respectively (Fig. 5b). However, the resultant genome-editing efficacy of HSPCs with these two approaches showed no statistical difference in both E- and G/M-colonies (Fig. 5c); although, a slight increase was found with Cas9/RNP method.

## Discussion

With the studies reported herein, we demonstrated a genome-editing approach to treat SCD. First, electroporation is free of viral and/or bacterial genes. Second, this approach requires only a small amount (2–3 mL) of the patient peripheral blood. Due to rapid destruction of sickled RBCs, the SCD patients always have anemia, which stimulates reactive proliferation of HSPCs, and thus, HSPCs from the peripheral blood are readily available. Moreover, our step-by-step validation studies, using both cultured cell model and primary HSPCs isolated from the patient blood, confirmed reproducibility of this CRISPR approach with a stable hemoglobin genome-editing efficacy of ~9% throughout all studies. Clinically, SCD is caused by homozygous *HbS/HbS* in patients; however, SCT carriers with *HBB/HbS* genotype do not suffer from the disease because HbA and HbS are co-expressed in RBCs [6]. This provides the cornerstone to cure the disease by genome-editing HSPCs from SCD to SCT genotype by applying the CRISPR/Cas9 approach we developed herein and the autologous transplantation of the genome-edited progenitor cells. In this context, pure genome-edited cells carrying SCT genotype are required for therapeutic purposes. To address the clinical needs and lay the foundation to treat SCD, in this study, the erythroid progenitor cells were cloned from single colonies of HSPCs and further amplified post CRISPR genome editing. Subsequently, the cloned cells were subjected to comprehensive validation studies, including genotyping, sequencing, hemoglobin expression, and cell function. Importantly, this study demonstrated for the first time that genome-editing *HbS* of patient HSPCs resulted in cellular function reinstitution of cloned offspring RBCs (Fig. 5c and Additional files 2, 3, and 4: Movies S1–S3). These findings prove the likelihood to treat SCD by autologous transplantation of the cloned erythroid progenitor cells post cell function validation (Additional file 1: Figure S1B). Need to note, individual with SCT can have sickling phenomena under extreme circumstances. Thus, correction of SCD patient HSPCs from *Hbs/HbS* to *HBB/HBB* genotype is still preferred in the future study.

In this study, two separate point mutations 58T and 69A/70A were introduced as tracking markers in *HbS* by CRISPR (Fig. 2). Due to these genetic hallmarks, the resultant genome-engineered HBB* was easily identified during sequencing. Importantly, the presence of two sequence hallmarks in HBB* ruled out the possibility of experimental contamination, unexpected genome variation, or artificial sequence mutations. Sequencing analysis demonstrated that these genetic hallmarks (58T and 70A) were always co-present and were detected in all genome-edited HSPC clones; although, the two mutation sites were more than 10 nucleotides apart. These findings indicate that it is possible to simultaneously edit two separate target sites in the same gene by the CRISPR/Cas9 approach, using a single pair of sgRNA and HDR template. Simultaneous genome editing of two target sites can be useful to treat diseases carrying two or more point mutation sites that are close to each other.

The genome-editing CRISPR approach with Cas9 mRNA and repeated electroporation showed stable and reproducible efficacy ranging between 8.5 and 9.6% in both cultured SCD cell model and primary patient HSPCs (Fig. 3, Additional file 1: Figures S11 and S12). To simplify this approach, CRISPR employing Cas9 protein and a single electroporation step was also studied. Results showed a significant increase in colony formation of the treated HSPCs due to fewer electroporation steps, but no statistical change in genome-editing efficacy (Fig. 5). Collectively, these findings suggest that Cas9 mRNA and Cas9 protein have a similar potential to perform CRISPR genome editing, and that the achieved ~9% efficacy likely is the limit of CRISPR/Cas9 combined with electroporation in patient HSPCs. To validate the designed genome editing of *HbS* in patient HSPCs, target sequencing analysis was performed. However, for patient safety, whole genome sequencing may be necessary to eliminate potential risk of any off-target gene effects caused by CRISPR/Cas9 [34–37]. In this study, we validated the amplification method of cloned erythroid progenitor cells from single HSPC colonies, which enabled the preparation of enough cellular genomic DNA for both genotyping and target sequencing. Notably, the purified cellular genomic DNA is appropriate for whole genome sequencing analysis, which allows precise selection of cloned erythroid progenitor cells carrying normal genome sequences and designed genome editing at gene site(s) of interest for clinical use. It is expected that the rapid advances in CRISPR/Cas9 technology will reduce and ultimately eliminate the risk of off-target gene effects, and CRISPR/Cas9 will become the standard method to cure genetic disorders in routine clinical practice.

## Conclusions

This study is an exploration of genome editing of SCD HSPCs.

## Additional files:

Additional file 1: Figure S1-S16. Function reinstitution of offspring red blood cells cloned from the sickle cell disease patient blood by a clinically practicable CRISPR/Cas9 method. (DOCX 4365 kb)

## Abbreviations

CRISPR: Clustered regularly interspaced short palindromic repeats
crRNA: CRISPR targeting RNA
*EGFP*: Enhanced green fluorescent protein gene
EPE: Erythroid progenitor expansion
EPO: Erythropoietin
ETM: Erythroid terminal maturation
*HBB*: Hemoglobin beta chain gene
HbS: Hemoglobin-S
HDR: Homology-directed repair
HPLC: High-performance liquid chromatography
HSPCs: Hematopoietic stem/progenitor cells
IGF-1: Insulin-like growth factor-I
RBCs: Red blood cells
SCD: Sickle cell disease
SCF: Stem cell factor
sgRNA: Single guide RNA
tracrRNA: Trans-activating crRNA
ZFN: Zinc-finger nuclease
