# Supplementary material for: Cellular function reinstitution of offspring red blood cells cloned from the sickle cell disease patient blood post CRISPR genome editing
Source: J Hematol Oncol. 2017 Jun 13;10:119. doi: 10.1186/s13045-017-0489-9 (PMC5470227; doi:10.1186/s13045-017-0489-9)
Supplement: Supplementary file 4 — Function reinstitution of offspring red blood cells cloned from the sickle cell disease patient blood by a clinically practicable CRISPR/Cas9 method. (DOCX 4365 kb) [file 13045_2017_489_MOESM1_ESM.docx]

**Function reinstitution of offspring red blood cells cloned from sickle cell disease patient blood by a clinically practicable CRISPR/Cas9 method**

**Additional file 1 - Supplemental Materials and Methods**

**Cellular genomic DNA preparation**

Genomic DNA of cultured HEL cells was prepared by using the Blood & Cell Culture DNA Mini Kit (Qiagen, Valencia, CA). Briefly, cultured cells (1x10^5^) were suspended in 200 µL PBS, 20 µL of proteinase K and 200 µL buffer AL from the Kit were added, and the cells were incubated for 10 min at 70°C. Genomic DNA was purified with a DNeasy mini column and eluted in 50 µL of deionized water. DNA purity and concentration were measured with a Nanodrop ND-1000 spectrophotometer (Fisher Thermo Scientific).

To establish standard curves for RT-PCR genotyping, genomic DNA from whole blood samples was also prepared with the Blood & Cell Culture DNA Mini Kit (Qiagen). Briefly, 20 µL of anti-coagulated blood was mixed with 20 µL of proteinase K, the total volume was adjusted to 220 µL with PBS, and 200 µL of buffer AL were added. The solution was thoroughly mixed by vortexing and then incubated for 10 min at 70°C. Genomic DNA of whole blood was purified with a DNeasy mini spin column and re-suspended in 50 µL of deionized water. DNA purity and concentration were measured with a Nanodrop spectrophotometer.

For sequencing analysis of cloned cells from single HSPC colonies, cellular genomic DNA was amplified with the REPLI-g Midi Kit (Qiagen). Briefly, about 500 cloned cells were collected, washed with PBS, treated with denaturing buffer for 10 min on ice, and terminated with stop solution. Genomic DNA was amplified using REPLI-g Mini DNA polymerase for 16 h followed by heating at 65°C for 3 min to inactivate DNA polymerase. Amplified DNA products were purified with the purification column included in the REPLI-g Midi Kit. The purity and concentration of amplified DNA were measured with a Nanodrop spectrophotometer.

**RT-PCR genotyping**

All the primers for DNA amplification and reporting probes were designed with the AlleleID software and synthesized by IDT; they are listed in Table S2. The ABI StepOne RT-PCR system was employed for RT-PCR assay. The purified cellular genomic DNA concentration was adjusted to 20-50 ng/µL, and the PCR reaction was carried out in a final volume of 25 µL comprising TaqMan master mix, TaqMan genotyping assay mix (containing specific primers and TaqMan MGB probes), nuclease-free water, and 5 µL of template DNA. The cycling conditions were 30 sec at 60 °C, 10 min at 95 °C, followed by 50 cycles for 15 sec at 95 °C, and 1 min at 60 °C. The same assay mixture, containing template DNA, was tested under the same conditions as a negative control.

For sequencing analysis of cloned cells from single HSPC colonies, cellular genomic DNA of hemoglobin gene was amplified by PCR using paired primers (HBB outer forward and HBB outer reverse) and Fusion High-Fidelity DNA polymerase (Thermo Fisher Scientific, Houston, TX). PCR was performed for 30 sec at 98 °C; then 30 cycles at 98 °C for 10 sec, 62 °C for 30 sec, 72 °C for 30 sec, followed by 10 min at 72 °C. The amplified DNA products were analyzed by gel electrophoresis (2% agar) and purified with the Qiaquick PCR Purification Kit (Qiagen). Sanger sequencing analysis of hemoglobin gene was performed using HBB inner forward primer at the Core Lab of Baylor College of Medicine. All primers are listed in Table S1.

**Preparation of sgRNA for genome-editing**

The sgRNA and targeting sequences on hemoglobin gene were designed using the online CRISPR Design Tool (<http://tools.genome-engineering.org>). The sgRNA was then prepared by two approaches, via either *in vitro* transcription or intracellular transcription.

To prepare DNA sequences encoding sgRNA *in vivo*, PCR was performed using PX458 plasmid vector (Addgene, Cambridge, MA) as a template, and paired universal U6 forward and gene-specific U6 reverse primers specific for hemoglobin gene or fluorescent protein gene (*EGFP*) green as shown in Table S3. The resultant DNA sequences encoding sgRNA *in vivo* driven by U6 promoter were purified for cell transfection and intracellular transcription according to a previous report([1](#_ENREF_1)).

In addition, sgRNA specific for hemoglobin gene was generated by an *in vitro* transcription method as shown in Figure S6. To this end, template DNA was initially prepared by two PCR steps([2](#_ENREF_2)). Briefly, the first PCR reaction was performed to adapt the sequence containing scaffold portion of sgRNA from PX458 plasmid vector using universal “scaffold forward” and “scaffold reverse” primers. The generated DNA products were then used as the template for the subsequent PCR reaction with “scaffold reverse” primer and gene-specific T7 promoter-containing forward primer (primer T7GTG forward and primers T7GAG forward-1 and T7GAG forward-2). The final PCR products were purified with QIAquick PCR Purification Kit (Qiagen) and quantified with a Nanodrop spectrophotometer. A mixture of ATP, CTP, GTP, UTP, reaction buffer, template DNA, and enzyme were heated at 37°C for 16 h to carry out *in vitro* RNA transcription, according to the instructions of MEGAscript Kit (Ambion, Austin, TX). Template DNA was then removed by TURBO DNase, and the resultant sgRNA products were purified using the Megaclear Kit (Ambion), and quantified with a Nanodrop spectrophotometer. All primers are listed in Table S4.

**Optimization of electroporation conditions for biocompatible cell transfection**

In this study we used vector-free electroporation for cell transfection to develop a safe genome-editing approach and eliminate viral and/or bacterial genes posing uncertainty risks. For optimization, hard-to-transfect HEL cells were employed as a study model. First, Cy3-labeled ssDNA sequences encoding sgRNA *in vivo* specific for *HBB* were generated by PCR reaction using primers Cy3-U6 forward and GAG U6 reverse (Table S3) and purified. HEL cells (1 x 10^6^) were suspended in 100 µL of Nucleofector Kit V solution (Lonza) containing different amounts of Cy3-labeled DNA sequences. Electroporation was then carried out in a cuvette, using the Nucleofector 2B device with the preprogramed protocol X-005. The electroporated cells were cultured for 48 h, and analyzed by flow cytometry. Cell transfection efficiency was quantified by the percentage (%) of fluorescent cells, arising from intracellular Cy3-labeled DNA products introduced by electroporation, as shown in Figure S3.

In addition, to optimize genome-editing capacity, the stably EGFP-expressing HEL cells were electroporated to transfect 10 µg of Cas9 mRNA (Trilink, San Diego, CA), and cultured for 6 h to produce Cas9 protein in transfected cells. Subsequently, the cells were exposed to a 2^nd^ electroporation step in the presence of different amounts of DNA sequences encoding sgRNA *in vivo* specific for *EGFP* gene. Cells were then cultured for 7 days to knock-down cellular *EGFP* gene. Changes in cellular EGFP expression were analyzed by flow cytometry, and the percentage (%) of cells with knocked-down *EGFP* gene was calculated as shown in Figure S4.

**Validation of RT-PCR genotyping of hemoglobin genes**

First, to establish a standard RT-PCR genotyping protocol for identification of normal *HBB* and SCD *HbS* genes, RT-PCR primers and TaqMan MGB probe-reporters (6FAM-RT-PCR and VIC-RT-PCR probes specific for *HBB* for *HbS*, respectively) were designed by Primer Express (Applied Biosystems, Carlsbad, CA) as shown in Table S2. The purified genomic DNA samples from peripheral blood of SCD patients and healthy people were mixed at different ratios (10 ng/µL final concentration). RT-PCR amplification was carried out in a final volume of 25 µL consisting of TaqMan master mix, TaqMan genotyping assay mix including primers and TaqMan MGB probe reporters, and 5 µL of mixed genomic DNA templates. RT-PCR was performed for 30 sec at 60°C, 10 min at 95°C, followed by 50 reaction cycles for 15 sec at 95°C and 1 min at 60°C. Target gene amplification was monitored by StepOne device (Applied Biosystems) and results were plotted using StepOne software (Applied Biosystems) as shown in Figure S5.

Secondly, to compare the genome-editing potential of DNA sequence encoding sgRNA *in vivo* and *in vitro* transcribed sgRNA, a second electroporation step was conducted([3](#_ENREF_3)). Briefly, 1x10^6^ HEL cells were suspended in 100 µL of Nucleofector solution and then electroporated in the presence of 10 µg Cas9 mRNA, as previously described. After culturing for 6 h, electroporation was repeated with an equal amount (6 µg) of sgRNA or DNA sequence encoding sgRNA *in vivo,* with 4 µg of homology directed repair (HDR) DNA template GTG 180nt #1 specific for hemoglobin gene (Table S5). After culturing for 7 days, cells were harvested, and RT-PCR genotyping assay was carried out. Resultant genome-editing rates were analyzed and compared. As shown in Figure S7, a higher genome-editing efficiency was obtained with sgRNA, thus it was chosen for further validation throughout this study.

In addition, to optimize the ratio of sgRNA to HDR template for genome-editing, HEL cells (1 x 10^6^) were initially electroporated with 10 µg Cas9 mRNA, as previously described. The 2^nd^ electroporation step was conducted with different ratios of sgRNA to HDR template (total amount 10 µg) as shown in Figure S8. Resultant genome-editing efficacy was detected by RT-PCR genotyping assay.

Moreover, to determine optimal sgRNA targeting sequences of hemoglobin gene and their corresponding HDR templates, a pair of sgRNA HbA Tar#1 and HDR GTG 180nt#1 and a pair of sgRNA HbA Tar#2 and HDR GTG 180nt#2 were prepared (Table S5). Following initial electroporation with Cas9 mRNA, the HEL cells were subjected to a 2^nd^ electroporation step in the presence of paired sgRNA (6 µg) and HDR (4 µg). RT-PCR genotyping assay revealed that the paired sgRNA HbA Tar#1 and HDR GTG 180nt#1 induced higher genome-editing efficacy (Figure S9), and thus were used throughout this study.

For further optimization, HDR templates with different lengths including GTG 180nt#1 and GTG 127nt, which cut the hemoglobin gene site asymmetrically, were synthesized (Table S5). Similarly, post initial electroporation, the HEL cells were subjected to the 2^nd^ electroporation with sgRNA HbA Tar#1 paired with HDR GTG 180nt#1 or HDR GTG 127nt, as shown in Figure S10. RT-PCR genotyping assay revealed that both HDR templates had nearly identical efficacy for gene editing. For this reason, HDR template 127nt was selected for this study due to the ease of synthesizing shorter oligonucleotides.

**Establishment of SCD HEL cell model for genome-editing validation study**

First, the wild type hemoglobin gene (*HBB/HBB*) from cultured HEL cells was tested by RT-PCR genotyping assay. The sequence was confirmed by Sanger analysis using primers HBB outer forward and HBB outer reverse for amplification, and primer HBB inner forward (Table S1), at the Sequencing Core Facility.

Subsequently, HEL cells (1 x 10^6^) were electroporated with 10 µg Cas9 mRNA cultured for 6 h as previously described. For the second electroporation, 6 µg sgRNA HbA Tar#1 and 4 µg HDR template GTG 127nt were used with Nucleofector Kit V and the program X-005 on Nucleofector 2B device. Notably, to distinguish between the induced genome-edited and natural gene alteration, a C to T point mutation at position 58 of *HbA* gene was introduced as shown in Figure S11B. To validate genome-editing efficacy, 200 HEL cells post second electroporation were suspended in 100 µL of complete culture medium, mixed with 10x volume of Methocult H4230 (StemCell), and then seeded in a 35 mm culture dish for colony formation. After culturing for two weeks, cells were cloned from individual single colonies and their genome-editing status was analyzed by genotyping and sequencing. The genome-editing efficiency percentage (%) was calculated as the ratio of cell colonies with *HBB/HbS* vs. total cell colonies.

To create SCD HEL cell model with homozygous *HbS/HbS*, the previously cloned HEL cells were subjected to repeated CRISPR/Cas9 genome-editing. Similarly, cells were cloned and their genome-editing status was analyzed. The *HbS/HbS* genome-editing status in SCD HEL cell model was confirmed by genotyping and sequencing (Figure S11).

**Genome-editing SCD HEL cell model to SCT genotype**

First, a DNA fragment containing T7 promoter and *HbS* gene-targeting sequence was generated using paired primers Scaffold forward/Scaffold reverse in initial PCR and paired primers T7GTG forward and Scaffold reverse in 2^nd^ PCR, as shown in Figure S6 and Table S4. The sgRNA specific for *HbS* gene was then produced by *in vitro* transcription. In addition, the HDR template GAA 127nt was synthesized by IDT (Table S5) to convert *HbS* gene to *HBB** gene by point-mutation of codon GTG encoding valine to GAA encoding glutamic acid as shown in Figure S12A. Similarly, the SCD HEL cells were exposed to two steps of electroporation for cell transfection of Cas9 mRNA and subsequent sgRNA and HDR template as previously described. Notably, for genotyping assay of *HBB** gene, the same RT-PCR primers in combination with a new pair of TaqMan MGB probe reporters were used (6FAM-RT-PCR probe *HBB** and VIC-RT-PCR probe *HbS*, Table S2). The resulting genome-editing status was confirmed by sequence analysis, and efficacy (%) was calculated (Figure S12B).

**Electroporation-mediated vector-free transfection and colony formation of HSPCs**

Expanded HSPCs (5-10 x 10^4^/sample) were suspended in 100 µL of solution of Human CD34 cell Nucleofector Kit (Lonza) in a cuvette for the 1^st^ electroporation with 10 µg Cas9 mRNA using Nucleofector 2B device and program U-008. For the 2^nd^ electroporation, *in vitro* transcribed sgRNA and HDR template for engineering *HbS* to *HBB** gene were introduced in HSPCs using the validated methods described previously for genome-editing SCD HEL cell model.

To identify genome-editing status in individual cells, electroporated HSPCs were suspended in 100 µL StemSpan SFEM II medium and mixed with 10x volume of Methocult H4030 containing erythropoietin (EPO). HSPCs were then seeded in a 35 mm culture dish and cultured for two weeks for colony formation. Morphologically, erythroid progenitor colonies (E-colonies) were red due to the produced cellular hemoglobin, but granular/monocytic progenitor colonies (G/M-colonies) appeared semi-transparent. The total numbers of formed E- and G/M-colonies were counted under a microscope.

**Single-step electroporation with Cas9 protein and RNA duplex**

First, RNA duplex was prepared by mixing 0.3 nmol of synthetic CRISPR RNA (crRNA) specifically targeting *HbS* and an equal amount of synthetic gene tracrRNA in 7 µL of nuclease-free Tris buffer (10 mM, pH 7.4). Subsequently, 1 µL of purified Cas9 protein (10 µg) was added to the RNA duplex and incubated for 10 min to form Cas9/gRNA ribonucleoprotein (RNP) complex (https://www.idtdna.com/pages/products/genome-editing/crispr-cas9). The formed Cas9/gRNA RNP complex was then mixed with 2 µL HDR template (4 µg) to convert *HbS* to *HBB**. In total, 10 µL of RNP and HDR template mixture was introduced by electroporation into HSPCs (5-10 x 10^4^/sample) in 100 µL of solution, as previously described. The crRNA sequences, which are specific for *HbS* gene, are listed in Table S6. Similarly, cells were cloned from single colonies and genome-editing status was detected by RT-PCR genotyping and target gene sequencing analysis. Results were compared to those obtained from the two-step electroporation method with Cas9 mRNA.

**Supplemental information - Supplemental Figures**

To develop a risk-free genome-editing approach, we initially tested electroporation method for intracellular delivery of CRISPR/Cas9 materials. To optimize intracellular delivery conditions, hard-to-transfect HEL cells were used (a human erythroleukemia cell line carrying normal *HBB/HBB* genotype)([4](#_ENREF_4)). To eliminate potential risks, entirely biocompatible Cas9 materials composed of synthetic oligonucleotides without viral and/bacterial genes were investigated. An optimal approach to achieve the highest efficacy of hemoglobin genome-editing was established through step-by-step validation.


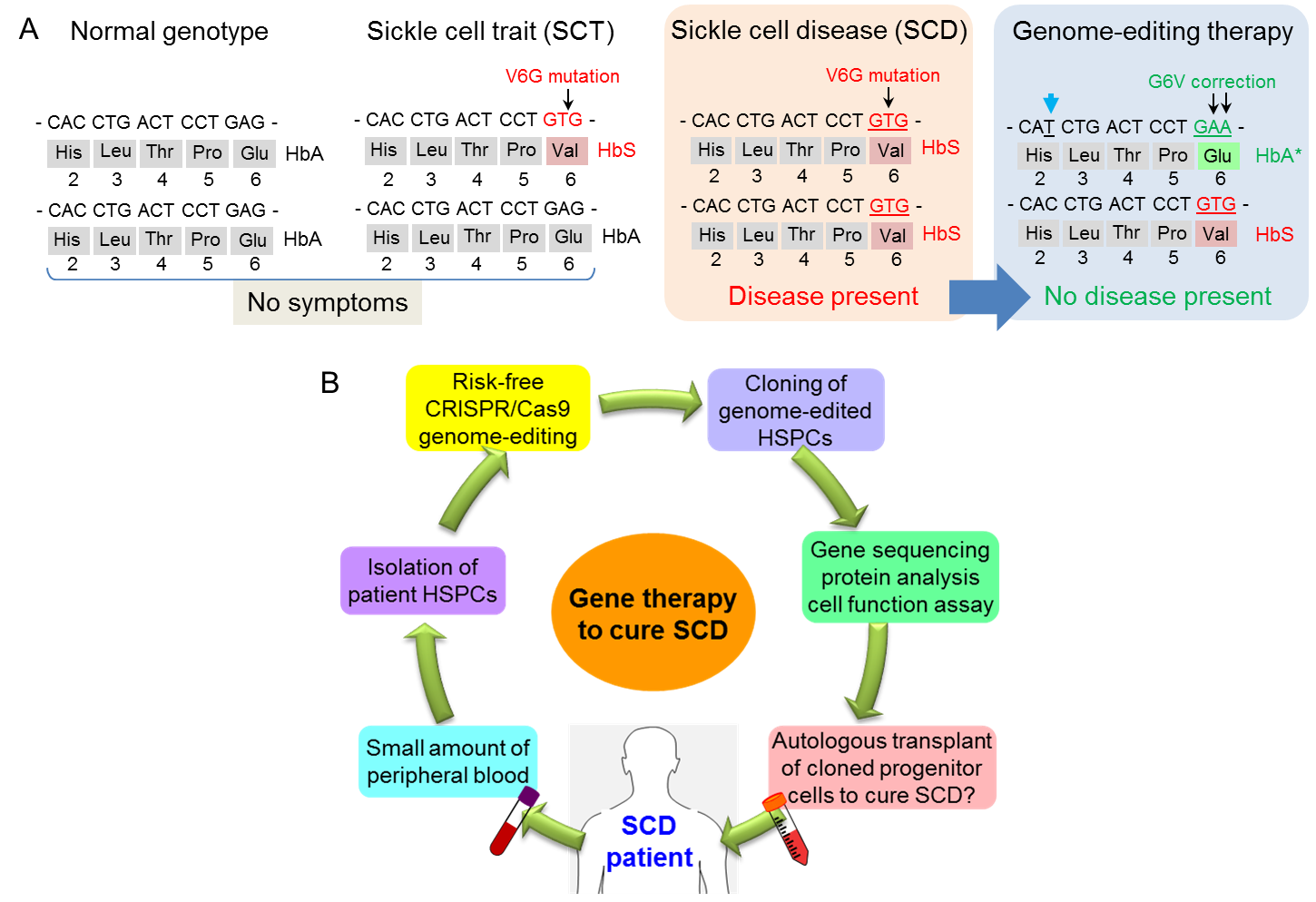


**Figure S1. Rationale and approach of genome-editing to cure SCD.** (A) Therapeutic rationale. Normal people carry *HBB/HBB* genotype; SCT carriers with *HBB/HbS* genotype usually do not have SCD symptoms. In SCD patients carrying *HbS/HbS* genotype, however, high levels of HbS are produced, and RBCs are deformed resulting in sickling cells under hypoxia conditions. Clinical findings provide a rationale to cure SCD disease by converting *HbS/HbS* to *HBB/HbS* genotype. (B) Therapeutic approach. The proposed clinically practicable CRISPR/Cas9 approach includes collection of SCD patient peripheral blood, HSPC isolation, risk-free CRISPR genome-editing, cloning of genome-edited HSPCs, and comprehensive validation of cloned erythroid progenitor cells. The expected goal is to cure SCD by autologous transplantation of the cloned genome-edited progenitor cells

**Figure S2. Preparation of DNA sequence encoding sgRNA *in vivo*.** The sgRNA sequences and the targeting sequences on hemoglobin gene were designed using the online CRISPR Design Tool (<http://tools.genome-engineering.org>). To generate DNA sequences encoding sgRNA *in vivo*, PCR was performed using PX458 plasmid vector as a template, and a pair of U6 forward and gene-specific U6 reverse primers for hemoglobin gene or fluorescent protein gene (*EGFP*) green as shown in Table S3. The resultant DNA sequences encoding sgRNAs *in vivo,* driven by U6 promoter, were purified.


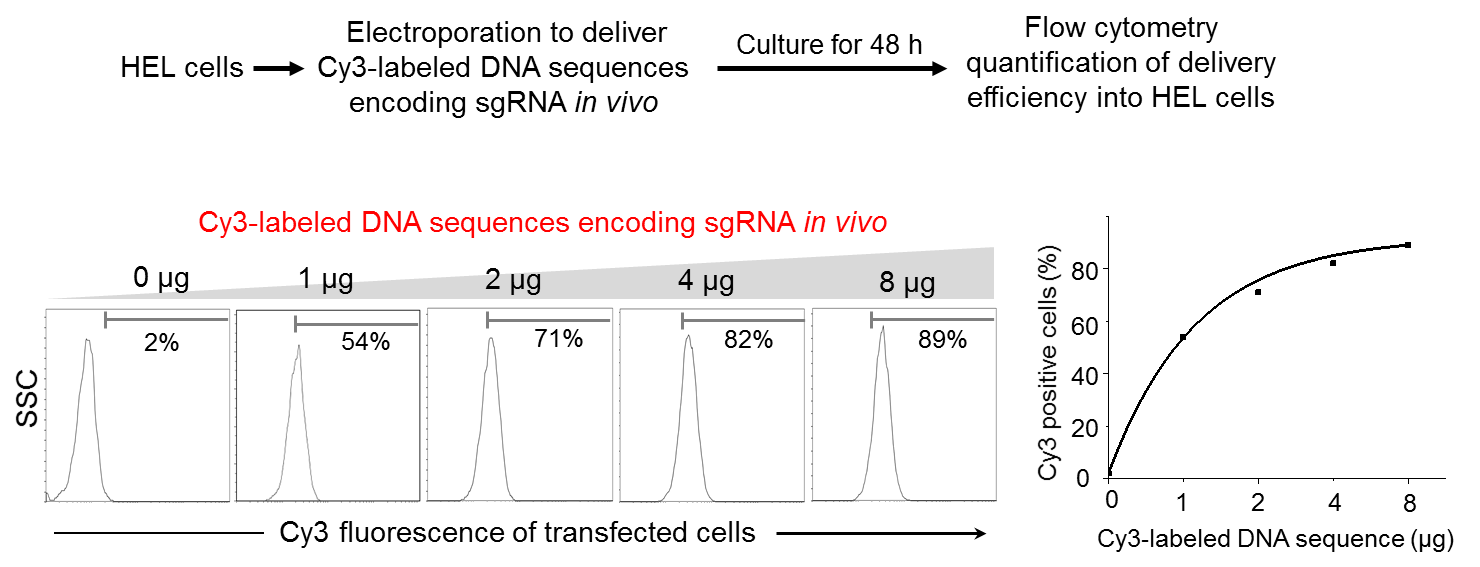


**Figure S3. Optimization of synthetic oligonucleotide delivery in hard-to-transfect HEL cells by electroporation.** For the purpose of validation, the DNA sequence encoding sgRNA *in vivo* specific for hemoglobin gene was generated and labeled with Cy3 fluorescent reporter. For intracellular delivery, HEL cells were subjected to electroporation in the presence of different amounts of Cy3-labeled DNA sequences. After culturing for 48 h, the HEL cells were harvested and analyzed by flow cytometry. Intracellular delivery efficiency of electroporation was then quantified by the percent (%) of fluorescent cells derived from intracellular Cy3-labeled DNA sequences. The highest efficacy (89%) of intracellular delivery was achieved in the presence of 8 µg of DNA sequence encoding sgRNA *in vivo*.


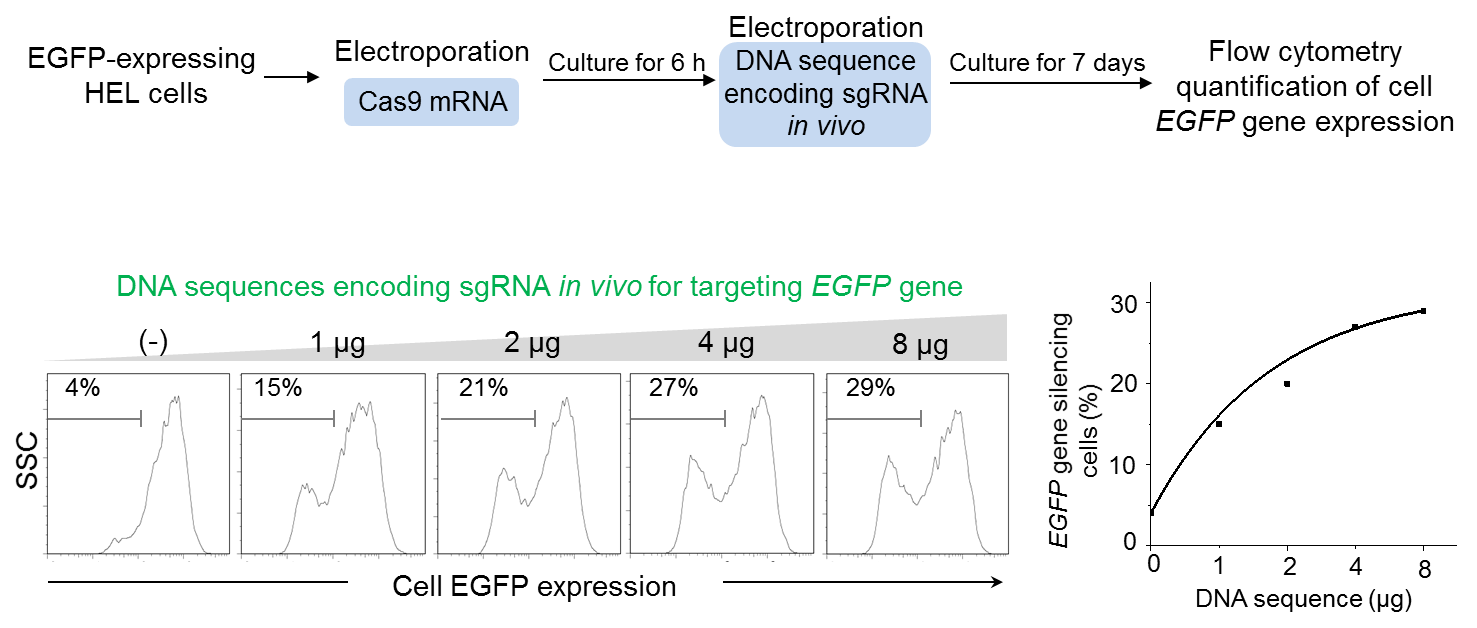


**Figure S4. Optimization of genome-editing efficiency of DNA sequence encoding sgRNA *in vivo***. For genome-editing, the stably EGFP-expressing HEL cells were initially subjected to electroporation for intracellular delivery of 10 µg Cas9 mRNA, and cultured for 6 h to express cellular Cas9 protein. Subsequently, cells were subjected to repeated electroporation in the presence of different amounts of DNA sequences encoding sgRNA *in vivo* specific for *EGFP* gene. Cells were further cultured for 7 days to silence cellular *EGFP* gene. Changes in cellular EGFP expression were quantified by flow cytometry, and the percent (%) cells with silenced *EGFP* gene was calculated. Genome-editing reached a plateau in the presence of 4 to 8 µg of DNA sequences encoding sgRNA *in vivo*, leading to the highest efficacy of 27-29% cells with *EGFP* gene silencing.


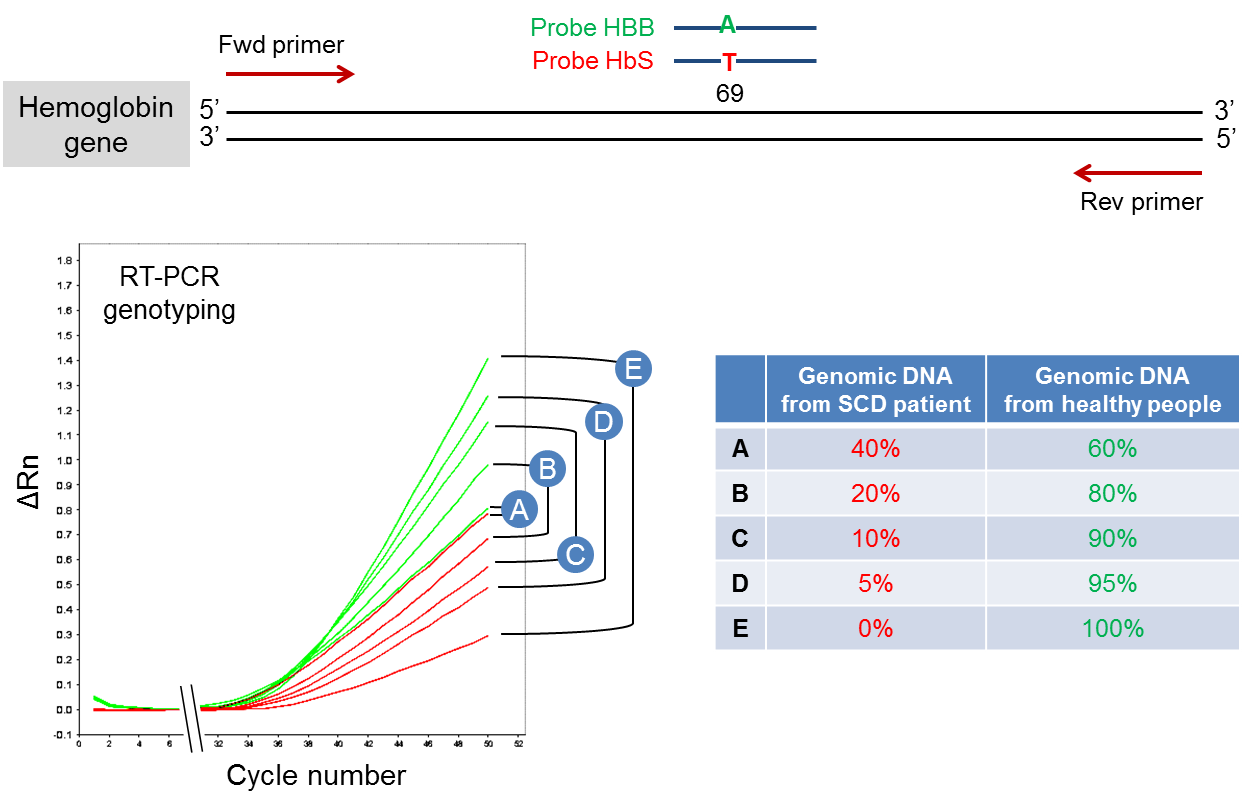


**Figure S5. Establishment of standard curves for TaqMan RT-PCR genotyping assay.** RT-PCR primers and TaqMan MGB probe-reporters were designed for genotyping of *HBB* and *HbS* as listed in **Table S2**. To establish standard curves, genomic DNA samples from peripheral blood of SCD patients and healthy people were purified, and mixed at different ratios as indicated. RT-PCR amplification was carried out and results were plotted using StepOne software.

**Figure S6. *In vitro* transcription of sgRNA.** (A) PCR amplification of DNA fragment encoding sgRNA scaffold was performed by using PX458 plasmid and paired “scaffold forward” and “scaffold reverse” primers. (B) The generated DNA fragment was used as the template for subsequent PCR with “scaffold reverse” and gene-specific T7 promoter-containing forward primers. (C) DNA products containing T7 promoter were used for *in vitro* transcription of sgRNA. All primers are listed in Table S4.


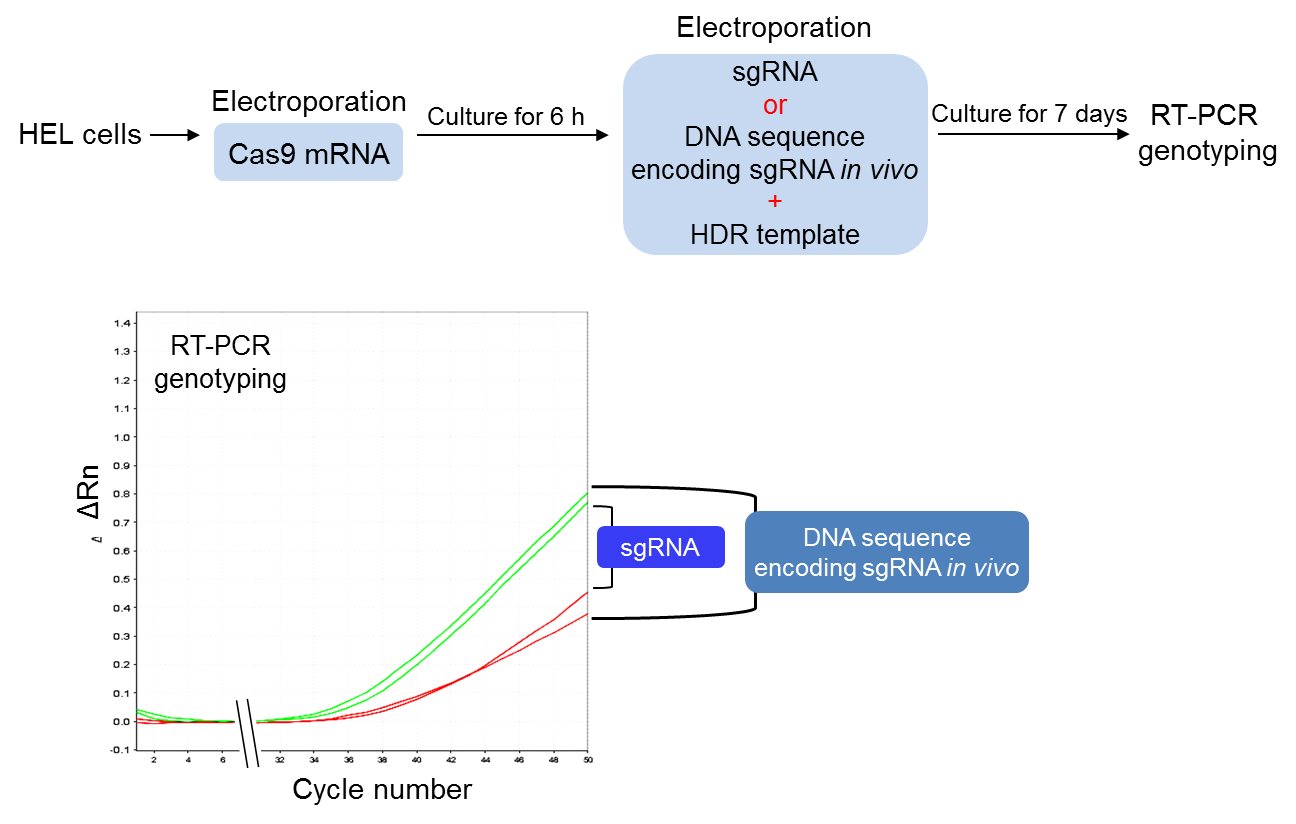


**Figure S7. Comparison of sgRNA and DNA sequence encoding sgRNA *in vivo* for CRISPR/Cas9 genome-editing**. HEL cells were initially subjected to electroporation for intracellular delivery of Cas9 mRNA. Subsequently, a second electroporation step was conducted in the presence of equal amounts of sgRNA or DNA sequence encoding sgRNA *in vivo* with HDR DNA template GTG 180nt #1 specifically targeting hemoglobin gene. RT-PCR genotyping of resultant cells indicated that sgRNA had slightly higher genome-editing potential than DNA sequence encoding sgRNA *in vivo*. Therefore, sgRNA was used in the following validation study.


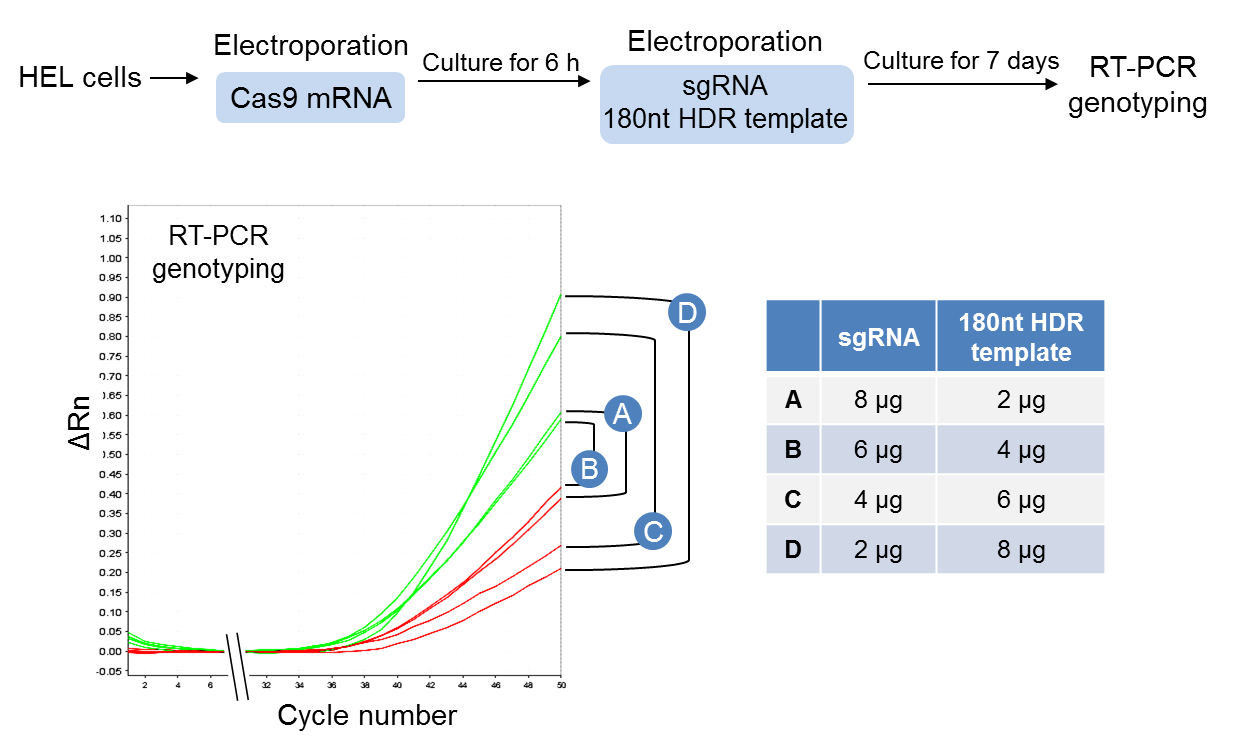


**Figure S8. Optimization of the ratio of sgRNA to HDR template for genome-editing.** After initial electroporation of HEL cells for intracellular delivery of Cas9 mRNA, electroporation was repeated in the presence of different ratios of sgRNA to HDR template (total amount 10 µg) as listed in the table. RT-PCR genotyping of resultant cells showed that the highest genome-editing efficacy was achieved with a ratio sgRNA/HDR template = 6 µg/4 µg, and this ratio was used in the following studies.


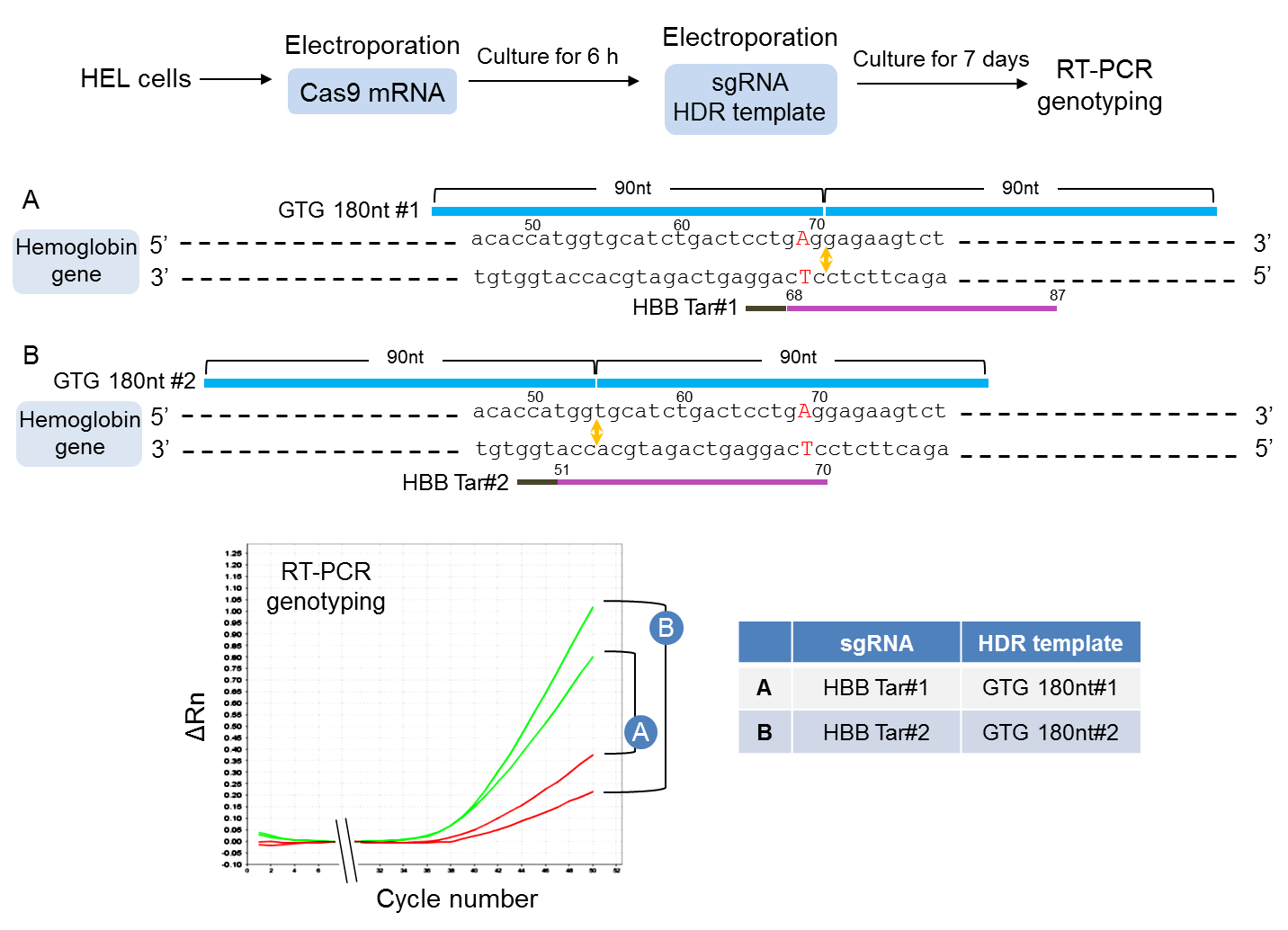


**Figure S9. Validation of different sgRNAs and HDR templates for genome-editing.** To determine optimal sgRNA targeting sequences on hemoglobin gene and their corresponding HDR templates, a pair of sgRNA HBB Tar#1 and HDR GTG 180nt#1 and a pair of sgRNA HBB Tar#2 and HDR GTG 180nt#2 were designed. Following initial electroporation of HEL cells for intracellular delivery of Cas9 mRNA, electroporation was repeated in the presence of paired sgRNA and HDR at their optimal ratio 6 µg/4 µg. RT-PCR genotyping of the resultant cells revealed that the paired sgRNA HBB Tar#1 and HDR GTG 180nt#1 achieved higher genome-editing efficacy, and thus were used in the following studies.


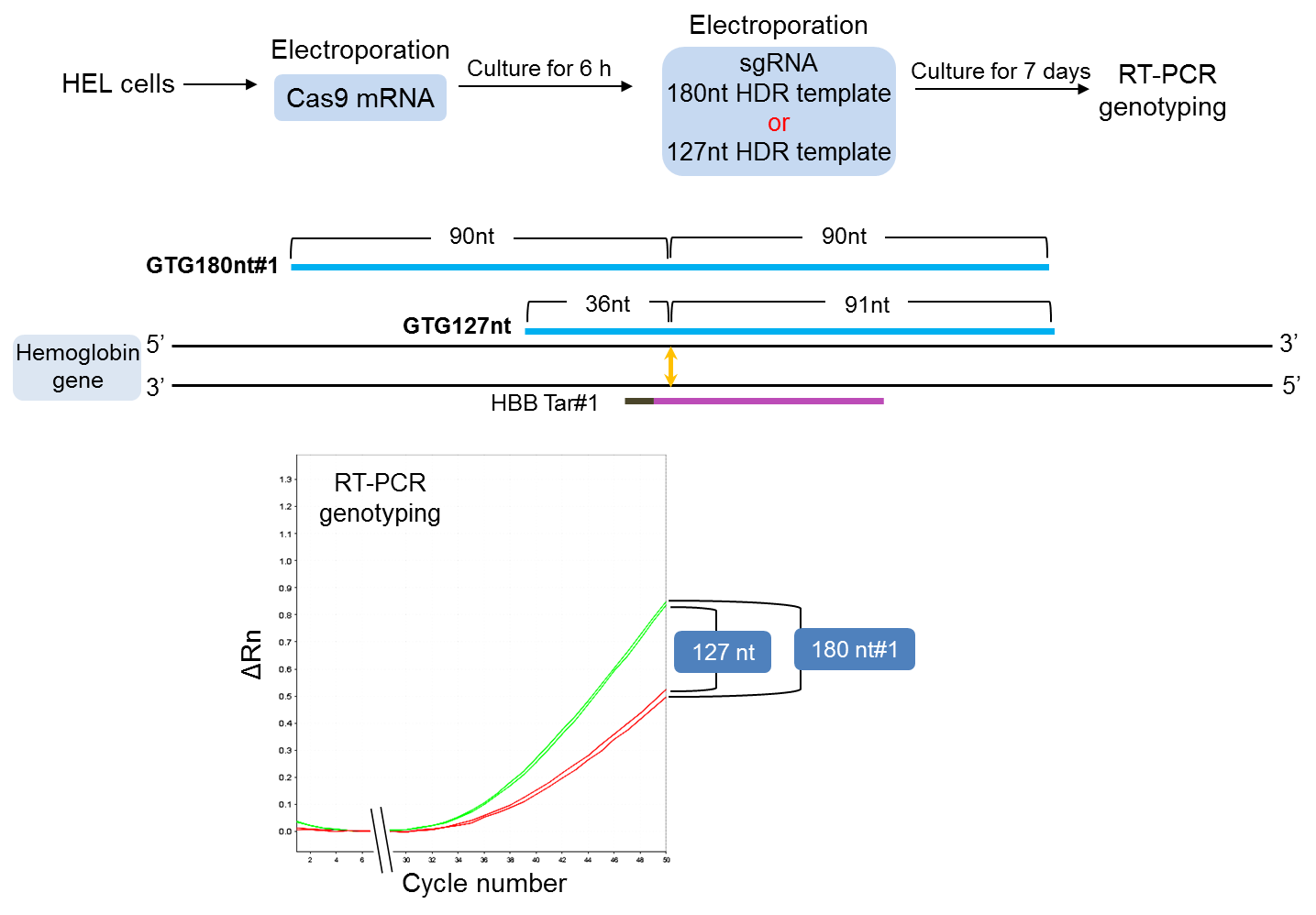


**Figure S10. Validation of HDR templates with different lengths for genome-editing.** HDR templates with different lengths (GTG 180nt#1 and GTG 127nt as shown in **Table S5**) were designed. Notably, HDR template GTG 127nt cut the hemoglobin gene site asymmetrically. Similarly, post initial electroporation, the HEL cells were subjected to a second electroporation step for intracellular delivery of sgRNA HBB Tar#1 paired with HDR GTG 180nt#1 or HDR GTG 127nt. RT-PCR genotyping assay revealed that both HDR templates had nearly identical efficacy for genome-editing. Because HDR template GTG 127nt was shorter than GTG 180nt#1 by 53 nt and easier to synthesize, it was selected for the following studies. After step-by-step optimization of all reaction conditions, a CRISPR approach employing electroporation and completely biocompatible Cas9 materials was established. For further validation, the developed CRISPR approach was used to create a SCD cell model. Subsequently, genome-engineering of SCD cell model was performed to convert *HbS* to *HBB,* and genome-editing efficacy was determined.


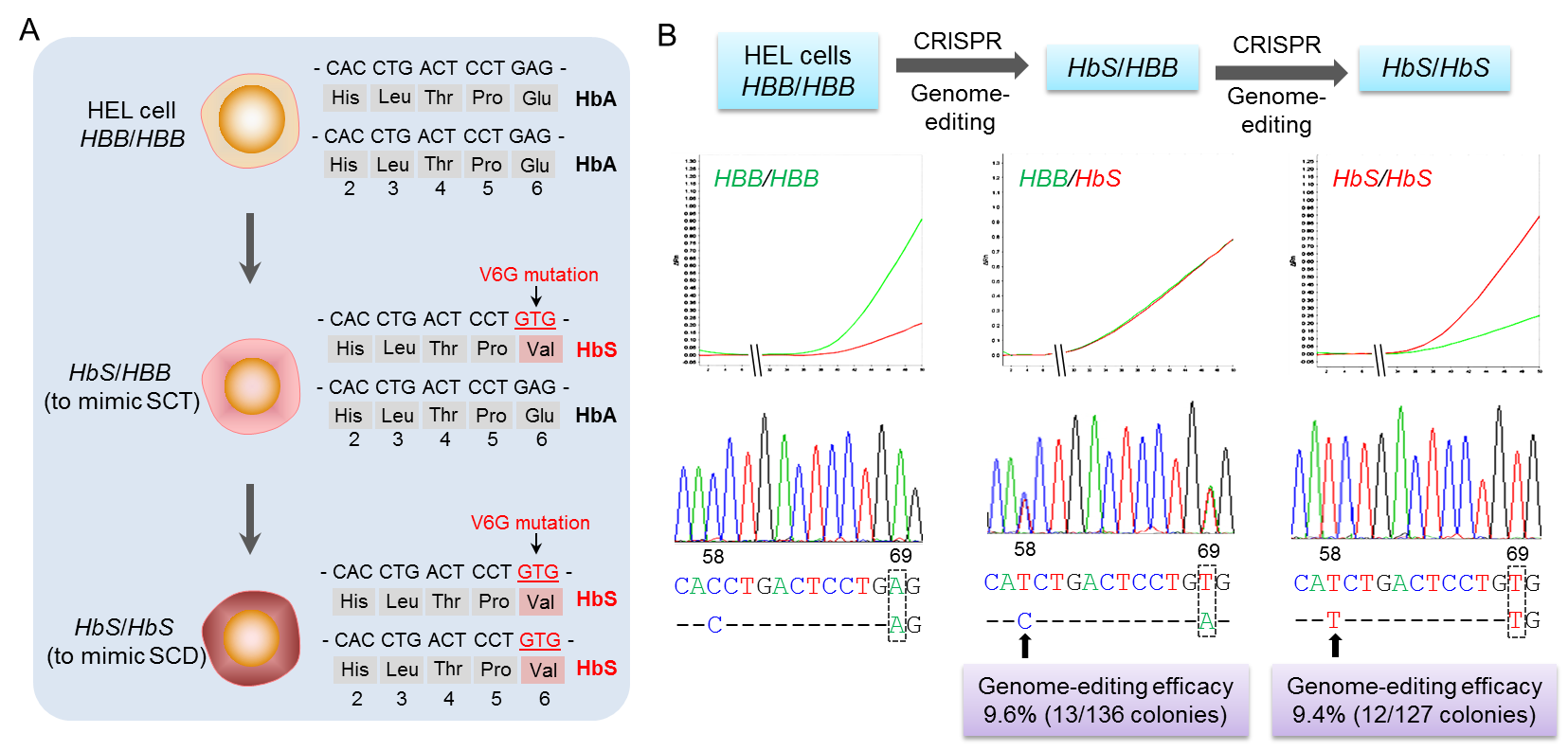


**Figure S11. Scheme for SCD cell model generation.** (A) Serial genome-engineering of the HEL cells was performed to convert *HBB/HBB* genotype to heterozygous *HBB/HbS*, and then to homozygous *HbS/HbS,* which represents genotype of SCD patients with V6G mutation in beta globin chain subunit of hemoglobin A protein (HbA)*.* (B) Generated cells were cloned at each step, and genome-editing status of individual cell colonies was examined by RT-PCR genotyping and sequencing analysis. For tracking purposes, 58T was introduced into sequence (marked with arrow); this had no impact on amino acid sequence of hemoglobin. In order to generate *HbS* genotype, 69T was introduced into sequence (boxed) resulting in V6G mutation in hemoglobin protein. Sequencing analysis of cloned cells revealed a stable genome-editing efficiency, namely 9.6% and 9.4% in each CRISPR step, respectively.


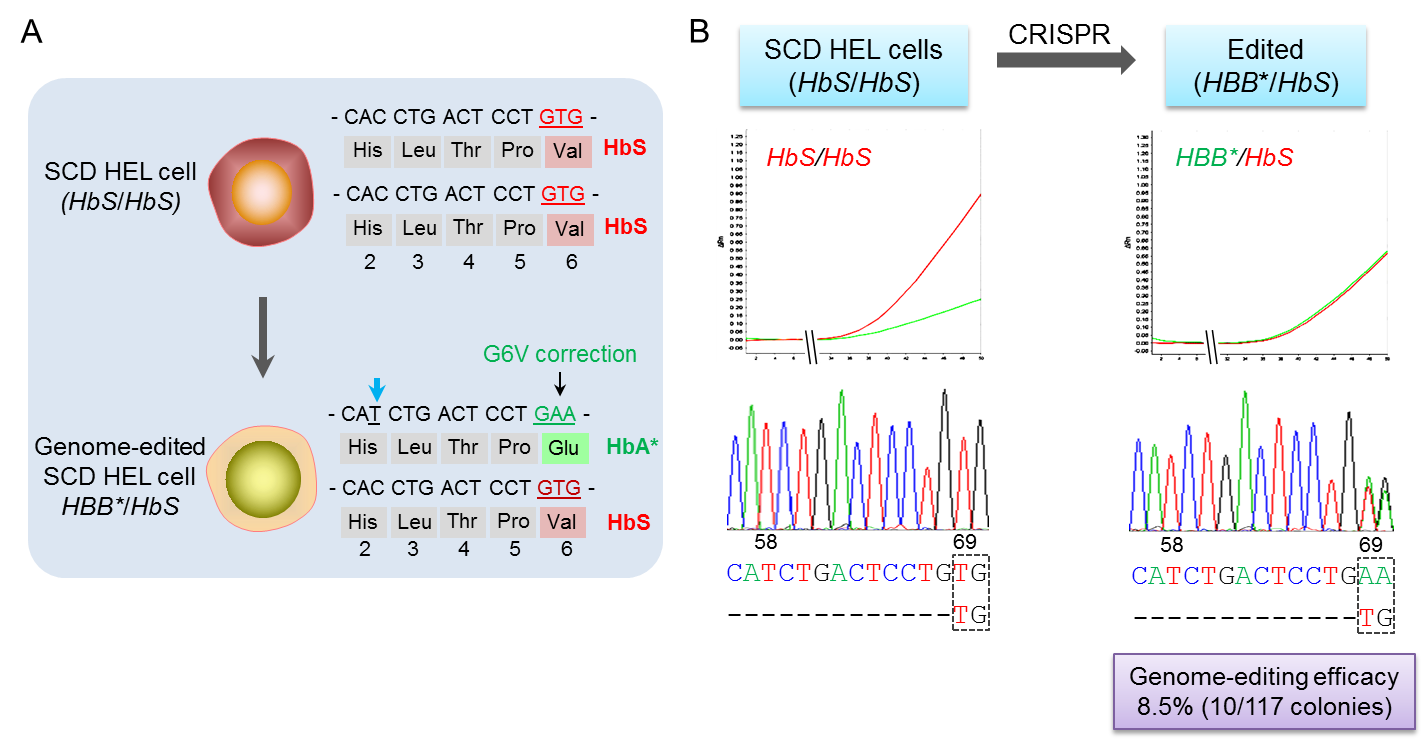


**Figure S12.** **Scheme for genome-editing of SCD cell model.** (A) To mimic gene therapy, genome-editing of SCD HEL cells was performed by converting *HbS/HbS* to *HbS/HBB* genotype (SCD to SCT genotype). (B) To achieve G6V correction, nucleotide 69T was substituted with 69A in *HbS*. In addition, to rule out experimental artifacts, 70A was introduced as an internal tracking marker to engineer *HBB** gene. The resultant HbA* protein was identical to natural HbA because introduction of 70A did not alter the amino acid sequence in hemoglobin beta globin chain. Both genotyping and sequencing analyses of resultant cell colonies confirmed 8.5% efficacy of genome-editing, which is similar to that observed in each step of cell model generation. These validation data demonstrated the reproducibility of the developed CRISPR/Cas9 approach for clinical studies using patient specimens.

**Figure S13. Chromatogram of Sanger sequencing from single colony of genome-edited SCD HEL cells.**

**Figure S14.** **Morphology of cells picked from the E-colonies.** Morphological examination of cells from 2 representative E-colonies was performed with cytospin and Wright Giemsa stain.


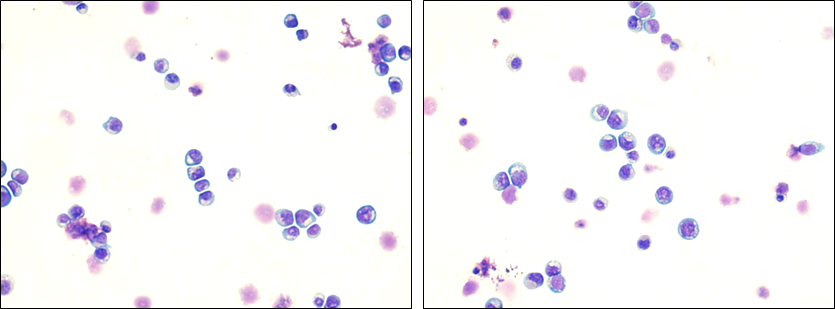


**Figure S15. Chromatogram of Sanger sequencing from single E-colony (A) and G/M-colony (B) of genome-edited SCD HSPCs.**

**A**

**B**

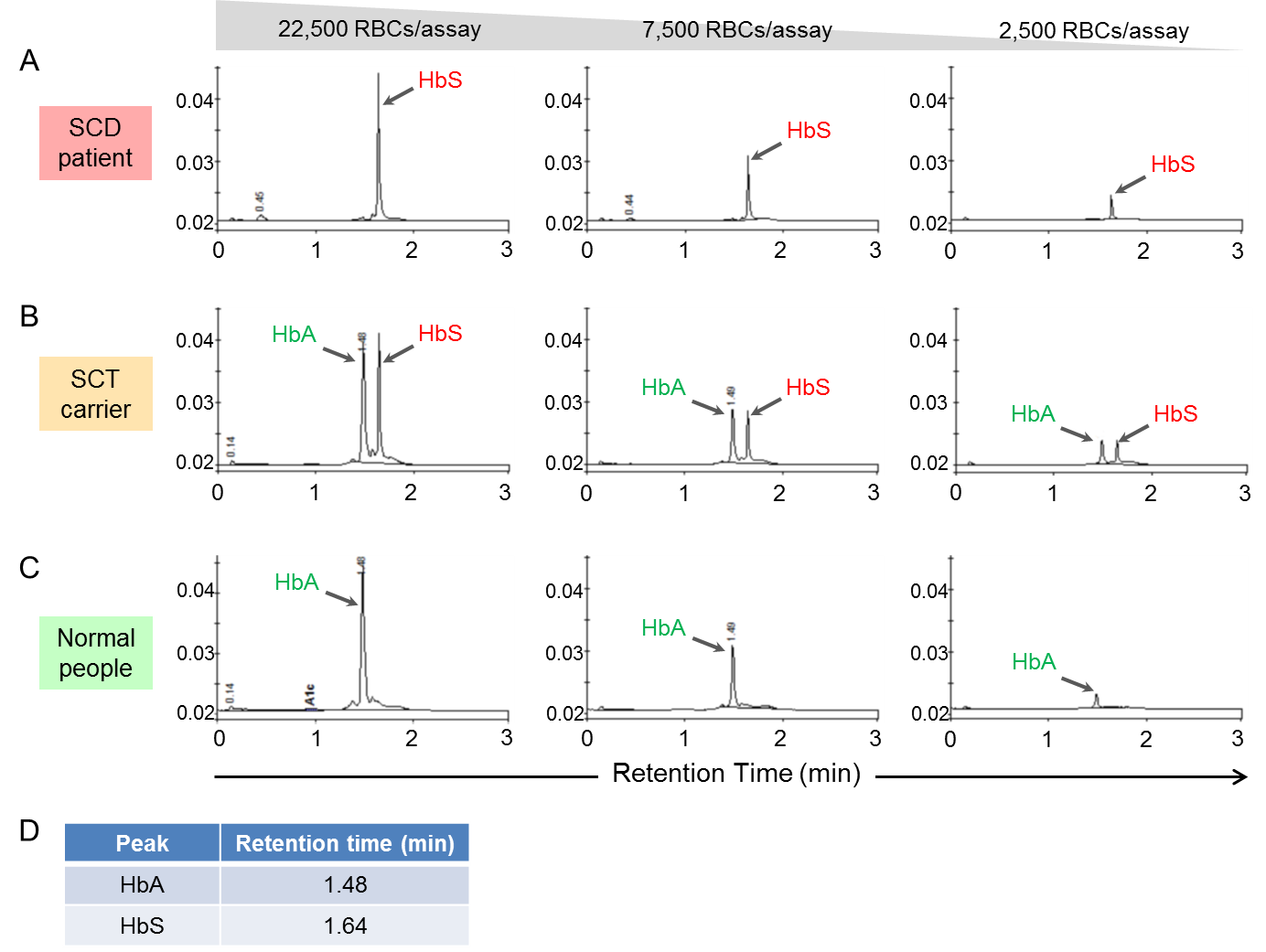


**Figure S16. Clinically viable HPLC assay to detect hemoglobin proteins in blood cells.** To validate a sensitive identification method of hemoglobin proteins, RBCs derived from peripheral blood samples were tested by a clinically viable HPLC assay as described in Materials and Methods. (A) SCD patient; (B) SCT carrier; (C) normal people; (D) HPLC retention times corresponding to peaks for HbA and HbS.

**Tables**

**Table S1.** Primers used for gene sequencing analysis.

**Table S2.** Primers and probes used for RT-PCR genotyping assay.


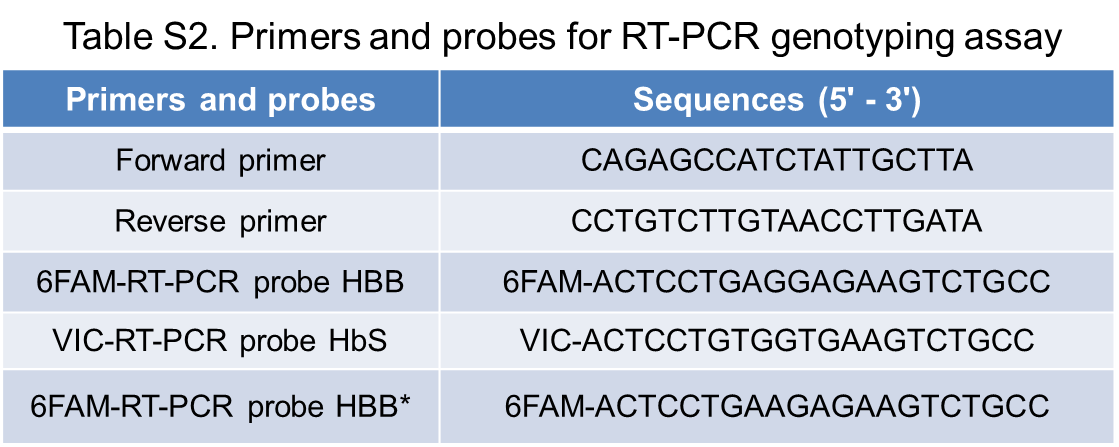


**Table S3**. Primers for generation of DNA sequence encoding sgRNA *in vivo*.

**Table S4.** Primers for *in vitro* transcription of sgRNA.

**Table S5.** The ssOND sequences of HDR templates.

**Table S6.** crRNA sequence specific for *HbS* gene


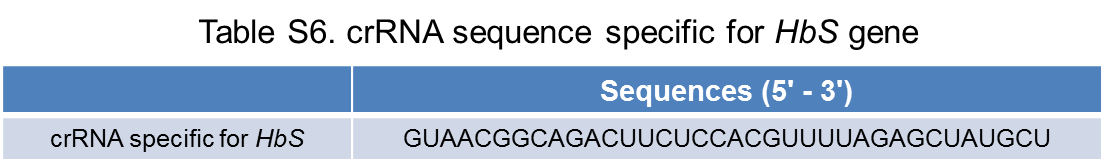


**Movies**

For cellular function study, *in vitro* differentiation of the cloned erythroid progenitor cells was conducted. Cell sickling assays were then performed as described in Materials and Methods. In addition, SCD patient blood was used as a standard control for cell sickling assay.

**Movie S1. Genome-edited RBCs.** Cell sickling assay of RBCs *in vitro* differentiated from cloned SCD patient erythroid progenitor cells, which carried *HBB*/HbS* genotype and expressed equal amount of HbA* and HbS proteins (Figure 4A, right panel), was performed as described in Materials and Methods. CRISPR genome-editing of patient HSPCs resulted in function reinstitution of the cloned offspring RBCs, which were resistant to hypoxia and didn’t display sickling formation. Cell sickling assay was recorded for 30 min at 1 min intervals. In the movie, 30 pictures are shown in total, and each picture is shown for 2 sec.

**Movie S2.** **RBCs that didn’t undergo genome-editing.** Cell sickling assay of RBCs *in vitro* differentiated from cloned erythroid progenitor cells, which did not undergo genome-editing and expressed cellular HbS protein only (Figure 4A, left panel). These cells were derived from HSPCs of the same patient and thus, were considered as an experiment internal control. Under the same hypoxia condition, sickle cell formation was observed in RBCs without genome-editing. Sickling assay was recorded for 30 min at 1 min intervals. In the movie, 30 pictures are shown in total, and each picture is shown for 2 sec.

**Movie S3.** **RBCs from SCD patient blood.** Cell sickling assay of RBCs from SCD patient peripheral blood was conducted as a standard control of the assay system. Sickle cell formation was observed in patient RBCs under the same hypoxia condition. Sickling assay was recorded for 30 min at 1 min intervals. In the movie, 30 pictures are shown in total, and each picture is shown for 2 sec.

1. Friedewald VE, Hare JM, Miller LW, Walpole HT, Jr., Willerson JT, Roberts WC. The editor's roundtable: advances in stem cell therapy for treatment of cardiovascular disease. Am J Cardiol. 2012 Sep 15;110(6):807-16.

2. Hoban MD, Cost GJ, Mendel MC, Romero Z, Kaufman ML, Joglekar AV, et al. Correction of the sickle cell disease mutation in human hematopoietic stem/progenitor cells. Blood. 2015 Apr 23;125(17):2597-604.

3. Hendel A, Bak RO, Clark JT, Kennedy AB, Ryan DE, Roy S, et al. Chemically modified guide RNAs enhance CRISPR-Cas genome editing in human primary cells. Nat Biotechnol. 2015 Sep;33(9):985-9.

4. Martin P, Papayannopoulou T. HEL cells: a new human erythroleukemia cell line with spontaneous and induced globin expression. Science. 1982 Jun 11;216(4551):1233-5.
